# Review of novel functions and implications of circular RNAs in hepatocellular carcinoma

**DOI:** 10.3389/fonc.2023.1093063

**Published:** 2023-02-20

**Authors:** Zheng Liu, Fangming Yang, Zhun Xiao, Yuexuan Liu

**Affiliations:** ^1^ Department of Combination of Traditional Chinese Medicine and Western Medicine, School of Medicine, Henan University of Chinese Medicine, Zhengzhou, China; ^2^ Department of Digestive Diseases, The First Affiliated Hospital of Henan University of Chinese Medicine, Zhengzhou, China

**Keywords:** circular RNAs, hepatocellular carcinoma, functions, implications, drug resistance, epigenetic modifications

## Abstract

Hepatocellular carcinoma (HCC) is one of the most frequent malignancies, with high incidence and mortality. As the majority of HCC patients are diagnosed at an advanced stage and die of recurrence and metastasis, its pathology and new biomarkers are needed. Circular RNAs (circRNAs) are a large subclass of long non-coding RNAs (lncRNAs) with covalently closed loop structures and abundant, conserved, stable, tissue-specific expression in mammalian cells. CircRNAs exert multiple functions in HCC initiation, growth and progression, serving as promising biomarkers for diagnosis, prognosis and therapeutic targets for this disease. This review briefly describes the biogenesis and biological functions of circRNAs and elucidates the roles of circRNAs in the development and progression of HCC, especially regarding epithelial-mesenchymal transition (EMT), drug resistance and interactions with epigenetic modifications. In addition, this review highlights the implications of circRNAs as potential biomarkers and therapeutic targets for HCC. We hope to provide novel insight into the roles of circRNAs in HCC.

## 1 Introduction

Hepatocellular carcinoma (HCC) is the most common and lethal malignancy worldwide. Overall, HCC morbidity ranks fifth of all malignancies, and HCC mortality ranks third in cancer-related death worldwide (1, 2). HCC has high mortality not only because of the high morbidity but also because of limited diagnostic measures and drug response. Moreover, most patients with HCC are diagnosed at an advanced stage, missing the opportunity for liver transplantation, resection or ablation. In advanced HCC, the response of patients with systemic treatment is limited, and drug resistance always occurs. Therefore, a deeper understanding of the molecular mechanism involved in HCC pathogenesis is necessary, and new biomarkers and effective therapeutic targets are urgently needed.

With the advancement of high-throughput RNA sequencing and bioinformatics, circRNAs have been identified as a subtype of lncRNAs with covalently closed-loop structures (3). Due to the specific structural lack of the 5’ cap and 3’ polyadenylated tails, circRNAs exhibited resistance to RNase R and showed a much longer half-life than the parental liner mRNA (48 h vs. 10 h) (4), showing better stability. In addition, circRNAs are expressed at 10 times higher levels than their corresponding mRNAs (5) and are more abundant than their parental mRNAs. Furthermore, circRNAs show high levels of conservation in diverse species and exhibit specific expression in different cells, tissues and developmental stages, enabling circRNAs to be promising biomarkers and therapeutic targets for disease (6–9). Although circRNAs have been identified as a subtype of lncRNAs and lncRNAs are longer than 200 nucleotides (10), the long range of circRNAs has not been definitively reported. As exonic circRNAs **(**ecircRNAs) account for the majority of circRNAs (>80%) (5, 11, 12), Zhang et al.’s research may provide some useful information. Most human endogenous circRNAs contain two or three exons, single-exon back-splicing requires 353 nucleotides in a median exon length, and multiple-exon back-splicing requires 112-130 nucleotides per exon (13). In addition to the full-length range, the nomenclature of circRNAs is also considered a problem. To date, no consensus circRNA nomenclature has been established. For example, circMTO1 ((hsa_circ_0007874, hg19: chr6:74175931–74,176,329) has at least 11 different names (14). Therefore, the nomenclature of circRNAs is quite ambiguous. Currently, several common naming methods are used, such as circDLC1 (15) and circSETD3 (16), which are named after the host gene. CircBase uses arbitrary numbers to name circRNAs (17).

CircRNAs have been demonstrated to play an essential role in the initiation and progression of multiple diseases, such as hypertension (18–20), cardiopathy (21), diabetes (22), and cancers (23–25). In hypertension, hsa_circ_0038648 has been found to be highly expressed in human aortic smooth muscle cells (HASMCs) and act as an independent risk factor for essential hypertension (18). In cardiopathy, circRNA_000203 aggravates cardiac hypertrophy by suppressing miR-26b-5p and miR-140-3p, leading to enhanced GATA-binding protein 4 (Gata4) levels (21). Moreover, circular RNA-ZNF532 was found to participate in diabetes-induced retinal pericyte degeneration and vascular dysfunction (22). In endometrial cancer, circRNA WHSC1 is upregulated, and overexpression of circRNA WHSC1 promotes the proliferation, migration and invasion and decreased apoptosis of endometrial cancer cells by targeting the miR‐646/nucleophosmin 1(NPM1) pathway (23). However, the role of circRNAs in HCC hepatocarcinogenesis and progression as well as their implications in HCC diagnosis and treatment remain to be elucidated.

In this review, we briefly present the formation and biological function of circRNAs and discuss their roles in proliferation, EMT, apoptosis, drug resistance, tumor metabolism, tumor immunology, interplay with epigenetic modification and novel applications, including as biomarkers and therapeutic targets, in HCC. We hope to provide novel insight into the roles and implications of circRNAs in HCC.

## 2 Overview of circRNA

CircRNAs are a unique group of long non-coding RNAs (lncRNAs) characterized by a single-stranded covalently closed-loop structure (3). CircRNAs were first identified in a virus in 1976 (3) and observed *via* electron microscopy in 1979 (26). CircRNAs were originally considered to be byproducts or abnormally spliced transcripts of mRNA (27–30). With the rapid development of high-throughput sequencing and bioinformatics, an increasing number of circRNAs have been verified to be involved in the initiation and progression of various diseases, such as cancers (23–25), hypertension (18–20), cardiopathy (21), and diabetes (22). Among these, the roles of circRNAs in cancers have attracted increasing attention.

## 3 Biogenesis and biological function of circRNA

**Figure 1:**
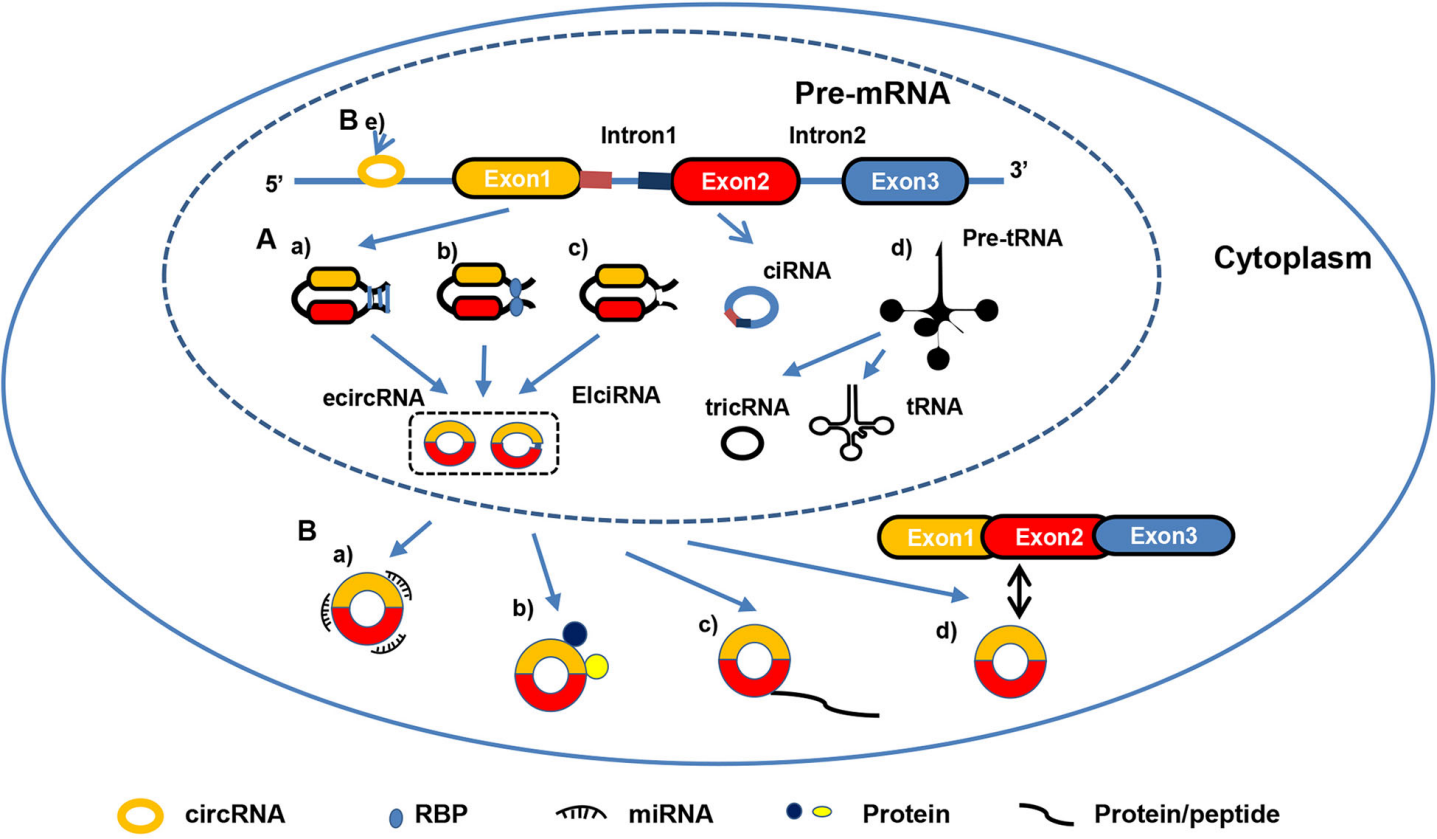
Biogenesis and functions of circRNA. **(A)** CircRNA biogenesis: **(a)** Intron pairing-driven circularization; **(b)** RBP-driven circularization; **(c)** spliceosome-dependent lariat-driven circularization; **(d)** TricRNA biogenesis by pre-tRNA intron splicing. **(B)** CircRNA functions: **(a)** Sponging miRNAs; **(b)** binding to proteins; **(c)** translating proteins/peptides; **(d)** competing with splicing of pre-mRNA; **(e)** binding DNA to regulate gene expression.

CircRNAs are formed through various circularizing mechanisms, such as RNA-binding proteins (RBPs) (31, 32), intron pairing (11, 33, 34), spliceosome-dependent lariat (35–38) and tricRNA splicing induced circularization (39, 40). Additionally, circRNAs can originate from diverse regions. CircRNAs are primarily classified into four categories based on their origin: exonic circRNAs (ecircRNAs) (5), exon−intron circRNAs (EIciRNAs) (41), circular intronic RNAs (ciRNAs) (42) and tRNA intronic circRNAs (tricRNAs) (39). Different circRNAs are present in different ratios, and various locations determine their biological functions. Several studies have shown that ecircRNAs, which account for >80% of identified circRNAs and are distributed predominantly in the cytoplasm, regulate gene expression by sponging microRNAs (miRNAs) or interacting with RBPs (11, 12). Unlike ecircRNAs, EIciRNAs and ciRNAs represent only a small fraction of circRNAs, and they are primarily located in nucleases and regulate the expression of their parental mRNAs (42). In general, circRNAs exert their functions in different ways, such as sponging miRNAs (43–45), interacting with proteins, including RBPs (12, 15, 46, 47), acting as transcriptional regulators (5, 41, 42) and competing with alternative splicing (48). In addition, the latest studies show that circRNAs have another unique feature, encoding peptides or proteins, even though they belong to “non-coding RNA” (49, 50) **(** **Figure 1** **).**

Due to the single-stranded covalently closed-loop structure, circRNAs possess enhanced resistance to ribonuclease (RNase), which results in much higher stability and abundance of circRNAs than miRNAs, lncRNAs and their linear counterparts (5, 51, 52). In addition, accumulating studies have reported that circRNAs have muscular tissue specificity and increased sequence conservation and show great potential as diagnostic and prognostic biomarkers and therapeutic targets in cancer.

## 4 Novel function of circRNA in HCC

Deregulation of circRNAs has been documented in many studies and plays an important role in the occurrence and development of HCC, including processes of proliferation, invasion, migration, apoptosis, drug resistance, tumor metabolism, and tumor immunity in HCC. In addition, circRNAs exert complex crosstalk with epigenetic modifications to participate in HCC progression.

### 4.1 Roles of circRNA in proliferation

Uncontrolled and unlimited proliferation is the most fundamental biological behavior of cancer cells. Evidence demonstrates that circRNAs play a crucial role in regulating the proliferation of HCC. A study conducted by Fu’s group showed that the circRNA MAN2B2 acts as an oncogene and promotes HCC proliferation. Fu and colleagues detected expression levels of circMAN2B2 in 32 HCC lesions and adjacent normal tissues and found it to be markedly up-regulated and that this up-regulation was related to clinical stage, implying its potential diagnostic value in HCC. Further research indicated that circMAN2B2 facilitates the proliferation of HCC by acting as a miR-217 molecular sponge and targeting mitogen-activated protein kinase 1 (MAPK1) (53). Another study employed microarray and qRT−PCR analyses to screen circSETD3 (hsa_circ_0000567) as a candidate circRNA for further study and discovered that circSETD3 acts as a tumor suppressor gene in HCC. Down-regulation of circSETD3 was correlates with poor prognosis and the clinical pathological characteristics of HCC. Further research revealed that circSETD3 exerts biological functions, such as suppressing proliferation and arresting the cell cycle, in HCC by targeting mitogen-activated protein kinase 14 (MAPK14) and mediated by miR-421 (16). Bioinformatics analyses accelerated the study of circRNAs and expanded RNA networks in HCC. Luo and colleagues analyzed three pairs of HCC tissue sample data from GEO datasets and found circCAMSAP1 to be apparently overexpressed in HCC tissues. Knockdown of circCAMSAP1 significantly restrained HCC cell proliferation, invasion and migration. In detail, circCAMSAP1 exerts its functions by sponging miR-1294 to increase GRAM domain-containing protein 1A (GRAMD1A) expression (54). Several other circRNAs, such as circ_0011385 and circ_0103809, were also found to participate in HCC proliferation (25, 55). Taken together, these findings suggest that circRNAs are crucially involved in HCC proliferation, contributing to hepatocarcinogenesis.

### 4.2 Roles of circRNA in EMT

**Figure 2:**
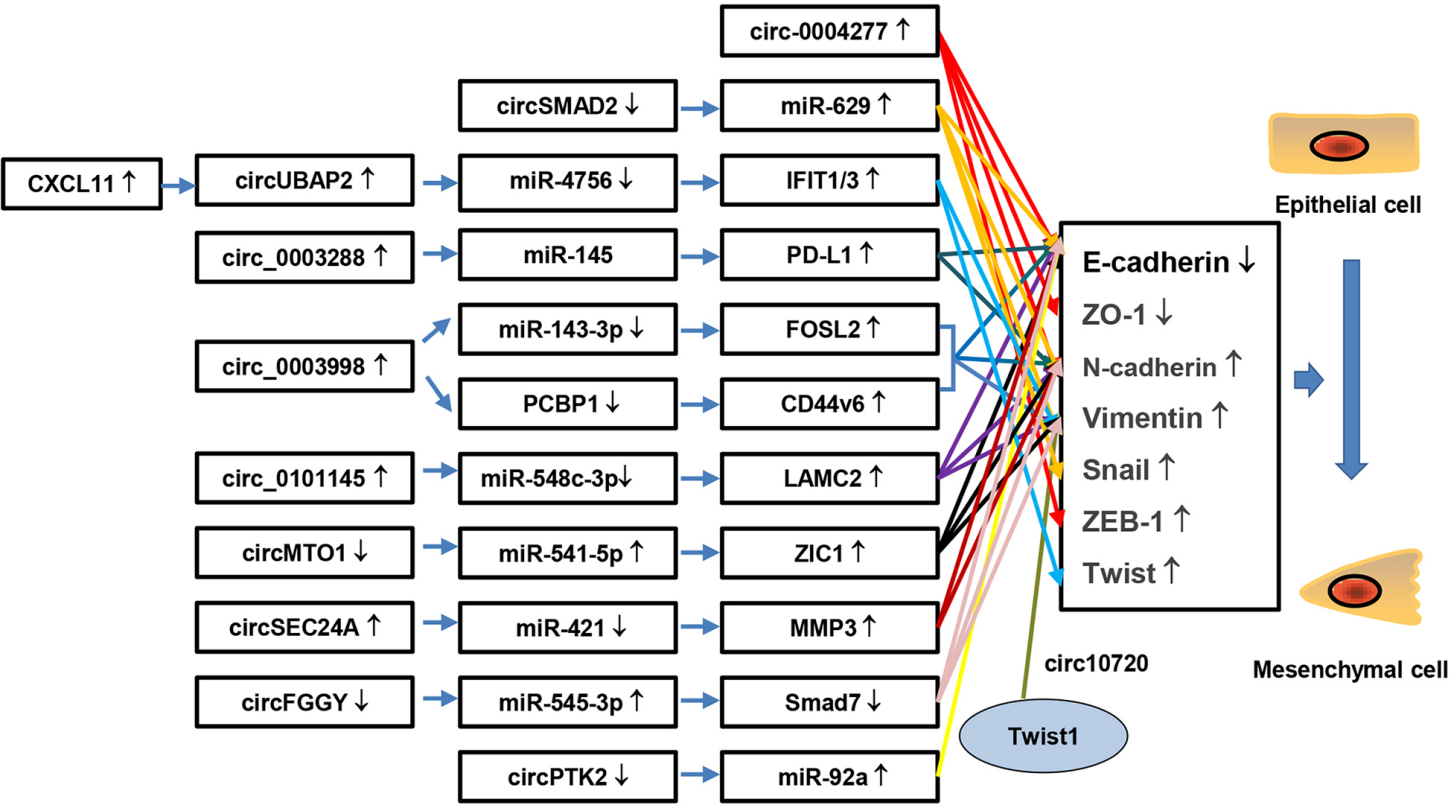
CircRNAs participate in EMT progression in HCC. CircRNAs participate in EMT by decreasing expression of epithelial phenotype markers, such as E-cadherin and ZO-1, while increasing that of mesenchymal phenotypic markers, such as N-cadherin and Vimentin.

The epithelial-mesenchymal transition (EMT) refers to a morphogenetic process by which epithelial cells lose their phenotype and acquire an mesenchymal phenotype (56, 57). EMT is essential for tumor mobility, invasion, metastasis and resistance to apoptosis stimuli. Moreover, EMT confers liver tumor cells with stem cell properties and marked therapeutic resistance (57). Therefore, EMT plays an important role in HCC metastasis and recurrence. In recent studies, several circRNAs have been found to participate in the development of EMT **(** **Figure 2** **)**. For example, circSMAD2 is obviously down-regulated in HCC cell lines and tissues, with the reduction correlating with the differentiation level of HCC tissues. Overexpression of circRNA SMAD2 suppresses the invasion, migration and EMT of HCC cells by reducing expression of N-cadherin, Snail and Vimentin, whereas it enhances E-cadherin expression. In-depth studies show that circRNA SMAD2 exerts biological functions by acting as a sponge of miR-629 (58). In this study, the authors demonstrated that circRNA SMAD2 modifies the EMT process by regulating biomarkers of EMT and sponging miR-629. However, the relationship between EMT biomarkers and miR-629 was not determined. Further research on this topic is warranted. Zhu’s research indicated that circ-0004277 acts as an oncogene in HCC by promoting EMT progression. Circ-0004277 expression is enhanced in HCC cells, tissues, and plasma exosomes. Overexpression of circ-0004277 in HCC cells was found to increase expression of ZEB1 and N-cadherin but decreased that of E-cadherin and ZO-1, resulting in promotion of EMT (59). In addition to directly regulate the expression of EMT biomarkers, circRNAs can regulate EMT through more complex mechanisms, and EMT biomarkers can regulate each other *via* circRNAs. Meng and colleagues found that Twist1 upregulates Cul2 circular RNA (circ-10720) expression by binding to the cullin2 (Cul2) promoter. Subsequently, elevated circ-10720 acts as a molecular sponge to absorb miRNA, including miR-490-5p, miR-1246, and miR-578, and target Vimentin. Regulated Vimentin activates EMT progression to facilitate HCC metastasis (60). Moreover, circRNAs can modulate the EMT process by regulating other proteins and multiple pathways rather than EMT biomarkers. Xu et al. found that circ-0003288 is overexpressed in HCC tissues compared to adjacent normal tissues and facilitates EMT, migration and invasion of HCC. An in-depth study indicated that circ-0003288 supports EMT, migration and invasion by sponging miR-145 to promote programmed death-ligand 1 (PD-L1) expression, activating the PI3K/AKT signaling pathway. In other words, hsa_circ_0003288 promotes EMT and invasion of HCC *via* the hsa_circ_0003288/miR-145/PD-L1 axis (61). Furthermore, Song et al. identified circRNA-0003998, which was found to be up-regulated in a portal vein tumor thrombus HCC group compared to the normal HCC group by RNA-sequencing. *In vivo* and *in vitro* experiments have shown that circRNA-0003998 promotes EMT in HCC. Mechanistically, researchers have revealed that circRNA-0003998 promotes expression of CD44v6 and EMT-related genes through direct binding to poly (rC) binding protein 1 (PCBP1) and acts as a competing endogenous RNA (ceRNA) of miR-143-3p, stimulating expression of FOS-like antigen 2 (FOSL2) to support EMT in HCC. In other words, circRNA-0003998 participates in HCC metastasis through two different axes: circ0003998/miR-143-3p/FOSL2 and circ0003998/PCBP1/CD44v6 (62). By regulating the miR-548c-3p/laminin subunit gamma 2 (LAMC2) axis, circ-0101145 acts as an oncogene to promote EMT in HCC (63). CircSEC24A (hsa_circ_0003528) and circFGGY also function as crucial regulators in EMT of HCC by competitively targeting miRNA, regulating target protein expression (64, 65).

These findings indicate that many circRNAs are involved in the process of EMT. Targeting circRNAs might block activation of EMT, further inhibiting invasion and metastasis and preventing recurrence of HCC. However, recent studies have mostly focused on the functions of circRNAs acting as sponges for miRNAs or proteins, and translation of circRNAs into proteins, peptides or pseudogenes has been less investigated. More research is needed.

### 4.3 Roles of circRNA in apoptosis

Apoptosis, which is generally known as programmed cell death, plays an essential role in restricting cell expansion, maintaining homeostasis and removing harmful cells. Limited apoptosis stimulates cell survival and drug resistance in cancer by modulating the tumor microenvironment (TME). Emerging studies have confirmed that circRNAs play a double-edged sword role in HCC apoptosis. Huang et al. discovered that hsa_circ_104348 is significantly up-regulated in HCC tissues and cells and facilitates HCC progression by promoting proliferation, migration, and invasion and suppressing apoptosis. Knockdown of hsa_circ_104348 suppressed hepatocarcinoma and lung metastasis. Furthermore, researchers identified that hsa_circ_104348 positively regulates expression of rhotekin-2 (RTKN2) and activates the Wnt/β-catenin signaling pathway by sponging miR-187-3p to promote HCC progression (66). In contrast to hsa_circ_104348, overexpression of circADAMTS13 induced apoptosis. Another study carried out by Qiu et al. distinguished 38 down-regulated and 4 up-regulated circRNAs in HCC tissues by RNA sequencing. CircADAMTS13, which is derived from exons 13-14 of the host gene ADAM metallopeptidase with thrombospondin type 1 motif 13 (ADAMTS13), was clearly down-regulated. It was further proven that dysregulation of circADAMTS13 correlates with the pathogenesis of HCC. Functional studies found that overexpression of circADAMTS13 strongly suppressed cell proliferation and induced apoptosis, showing an intensive tumor suppressor effect on HCC progression (67). Collectively, these findings support the notion that circRNAs have a pivotal effect on apoptosis in HCC and that different circRNAs may exert opposite roles in apoptosis.

### 4.4 Roles of circRNA in drug resistance

In addition to surgery, transplant and intra-arterial therapies, systemic treatment, which mainly includes chemotherapeutics, targeted therapy, and immune checkpoint inhibitors, remains an essential part of HCC treatment (68). Indeed, systemic treatment appears to improve the overall survival and quality of life of HCC patients. Nevertheless, effective systemic treatment is severely obstructed by drug resistance. Interestingly, several circRNAs have been found to participate in HCC drug resistance and play a major role in this process (**Table 1**).

**Table 1:** Summary of circRNAs in drug resistance in HCC.

Drug name	CircRNA	Roles of circRNA in drug resistance	Signalling pathway	Reference
Sorafenib	hsa_circ_0058124 (circFN1)	Promoting sorafenib resistance	CircFN1/miR-1205/E2F1	(70)
	circRNA-SORE	Sustaining sorafenib resistance	circRNA-SORE/miR-103a-2-5p and miR-660-3p/Wnt/β-catenin pathway	(71)
	circFOXM1	Sustaining sorafenib resistance	circFOXM1/miR-1324/MECP2	(72)
	circ102049	Reducing sorafenib sensitivity	Circ102049/miR-214-3p/RELN axis	(73)
	circUBE2D2	Reducing sorafenib sensitivity	circUBE2D2/miR-889-3p/LDHA	(74)
Lenvatinib	circMED27	Promoting lenvatinib resistance	circMED27/miR-655-3p/USP28	(75)
Anti-PD1 therapy resistance	circMET	Promoting anti-PD1 therapy resistance	circMET/miR-30-5p/snail/DPP4/ CXCL10 axis	(76)
	circTMEM181	Promoting anti-PD1 therapy resistance	circTMEM181/miR-488-3p/CD39/ CD73/eATP-adenosion pathway	(77)
Oxaliplatin	circFBXO11	Promoting oxaliplatin resistance	circFBXO11/miR-605/FOXO3/ABCB1 axis	(78)
Cisplatin	circ-0031242	Promoting cisplatin resistance	circ-0031242/miR-924/POU3F2 axis	(79)
	circRNA-101505	Enhancing the sensitivity to DDP	CircRNA-101505/miR-103/NOR1	(80)
Adriamycin	circFoxo3	Sustaining adriamycin resistance	circFoxo3/miR-199a-5p/ABCC1 axis	(81)

Targeted therapy has been a hot topic of cancer treatment for several years. Sorafenib was the first target drug approved by the US Food and Drug Administration (FDA) for advanced liver cancer, prolonging the median survival and progression time by nearly 3 months (69). Unfortunately, development of drug resistance to sorafenib is becoming increasingly common. Accumulating evidence has shown that a large number of circRNAs are involved in sorafenib resistance. For example, Yang et al. found 416 markedly up-regulated circRNAs and 332 obviously down-regulated circRNAs in sorafenib-resistant HepG2 cells compared with normal HepG2 cells. Subsequently, Yang and colleagues verified hsa_circ_0058124 (circFN1) as the most up-regulated circRNA in SR-HepG2 cells. Further studies revealed that circFN1 facilitates the progression of drug resistance to sorafenib by sponging miR-1205 to elevate E2F transcription factor 1 (E2F1) expression (70). Xu et al. reported that expression of circRNA-SORE is elevated in HCC cells and that this up-regulation is indispensable for sustaining sorafenib resistance. Knocking down circRNA-SORE enhanced the efficiency of sorafenib in HCC by promoting apoptosis. Mechanistically, circRNA-SORE sponges miR-103a-2-5p and miR-660-3p, activating the Wnt/β-catenin pathway to maintain sorafenib resistance (71). Moreover, circFOXM1 was found to sustain sorafenib resistance by modulating methyl-CpG binding protein 2 (MECP2) expression by targeting miR-1324 (72). In addition, circ102049 and circUBE2D2 are involved in sorafenib resistance. Circ102049 mediated sorafenib resistance *via* the miR-214-3p/reelin (RELN) axis, and circUBE2D2 reduces sorafenib sensitivity by sponging miR-889-3p, thereby regulating mRNA expression of lactate dehydrogenase A (LDHA) (73, 74). Therefore, we can infer that large numbers of circRNAs are abnormally expressed in sorafenib-resistant HCC. Some of them have been well studied in function and mechanism. However, the function and mechanism of circRNAs in sorafenib resistance are still unclear. Lenvatinib is another multitargeted tyrosine kinase inhibitor (TKI) drug recommended for advanced HCC in recent years. However, lenvatinib was also found to induce drug resistance, and circRNAs were involved. According to a recent study, circMED27 promotes lenvatinib resistance in HCC by targeting miR-655-3p and up-regulating ubiquitin-specific protease 28 (USP28). However, knockdown of circMED27 increases the sensitivity of HCC cells to lenvatinib (75). Taken together, many circRNAs are involved in sorafenib and lenvatinib resistance, promoting and sustaining drug resistance. Regardless, research on resistance to other targeted drugs, such as atezolizumab and bevacizumab, is limited. The main reason might be that the short time that the two drugs have been recommended as first-line treatment for HCC, which led to the cause of associated drug resistance had not been identified thus far.

Chemotherapy is an important part of comprehensive treatment for hepatoma carcinoma. The 2021 NCCN Clinical Practice guidelines recommend oxaliplatin + 5-fluorouracil + calcium folinate (FOLFOX) as the first-line option. Of course, other chemotherapeutic drugs, such as cisplatin and adriamycin, have also been used in HCC treatment. Unfortunately, there is extensive resistance to chemotherapeutic drugs, and circRNAs are involved. Li et al. determined that circFBXO11 is involved in oxaliplatin resistance *via* the miR-605/FOXO3/ATP binding cassette transporter subfamily B member 1 (ABCB1) axis (78). Silencing of circ-0031242 mitigates cisplatin (DDP) resistance and inhibits the proliferation, invasion, and migration of DDP-resistant HCC cells through the miR-924/POU class 3 homeobox 2 (POU3F2) axis (79). CircRNA-101505 enhances the sensitivity of HCC cells to DDP by sponging miR-103 and activating downstream oxidored-nitro domain-containing protein 1 (NOR1) (80). In addition, Huang et al. observed that circFoxo3 sustains adriamycin resistance by regulating the miR-199a-5p/ATP binding cassette subfamily C member 1 (ABCC1) axis in HCC (81). Therefore, drug resistance is widely present in the treatment of various chemotherapy drugs, which seriously affects the effect of chemotherapy treatment. CircRNAs participate in the resistance of chemotherapy drugs and can be used as a potential therapeutic target for chemotherapy resistance. However, there are few studies on oxaliplatin and 5-fluorouracil-related circRNAs. The role of circRNAs in the chemoresistance of HCC remains unclear. Therefore, more research is needed.

Immune checkpoints are an important component of tumor immunosuppression (82). Dysregulation of immune checkpoint expression helps tumor immune surveillance (83). Recently, targeting immune checkpoints has shown excellent therapeutic effects in cancer. However, with the clinical application of immune checkpoints, an increasing number of drug resistances have also been found. Moreover, circRNAs have been found to participate in immune checkpoint inhibitor resistance in multiple ways. Huang et al. demonstrated that circMET is overexpressed in HCC tissues and enhances EMT, invasion and metastasis of HCC cells. Hep1-6 cells with high circMET expression reduce the density of tumor-infiltrating CD8+ lymphocytes compared with parental normal Hep1-6 cells, inducing immunosuppression in mice. In detail, circMET led to immunosuppression and anti-programmed death 1 (PD1) therapy resistance by activating the miR-30-5p/snail/dipeptidyl peptidase 4 (DPP4)**/**CXCL10 axis (76). Another study showed that high expression of circTMEM181 correlates with anti-PD1 resistance. In detail, high exosomal circTMEM181 targets miR-488-3p and promotes CD39 expression in macrophages. Up-regulated CD39 binds with CD73, activating the eATP-adenosion pathway to result in anti-PD1 resistance in HCC (77).

**Figure 3:**
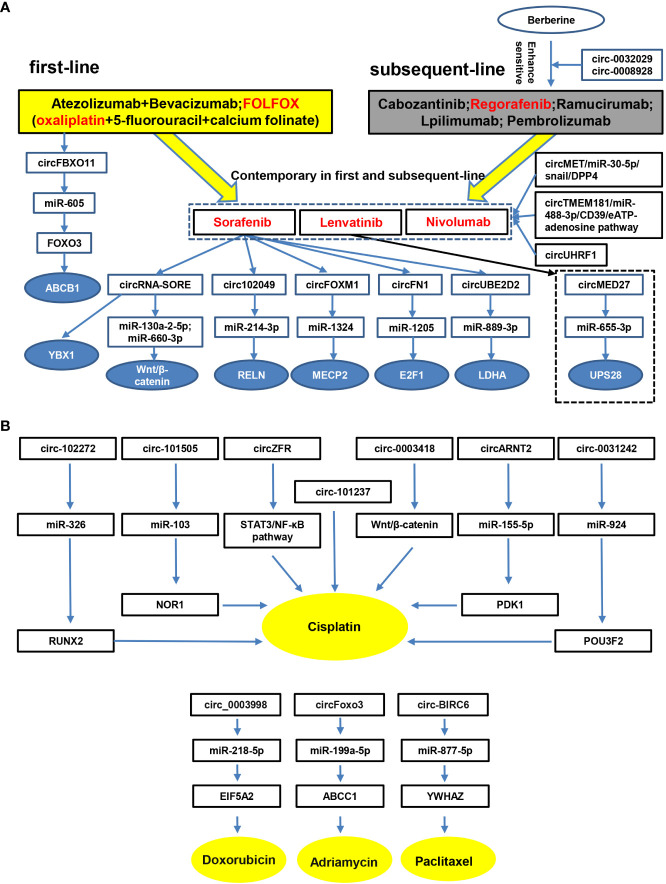
CircRNAs are involved in therapeutic resistance in HCC. Accumulating studies have demonstrated that circRNAs participate in therapeutic resistance in HCC. **(A)**, drugs in the first-line, subsequent-line and contemporary stages are listed according to NCCN Guidelines for Hepatobiliary Cancers 2021. Moreover, circRNAs and possible pathways involved in HCC therapeutic resistance are identified. **(B)**, complementary drugs and circRNAs related to therapeutic resistance in HCC, such as cisplatin, doxorubicin, adriamycin and paclitaxel, are listed.

Taken together, these findings provide strong evidence that circRNAs are involved in the drug resistance of HCC, and knock-down or exogenous overexpression of circRNAs might reverse this resistance. Therefore, circRNAs play a major role in drug resistance and may have great potential as therapeutic targets for HCC drug resistance. Furthermore, we summarize the circRNAs involved in the drug resistance of systemic therapies in HCC according to the NCCN Guidelines for Hepatobiliary Cancers 2021, an important reference for liver and gallbladder tumor screening, diagnosis, staging, treatment and management (**Figure 3A**). Resistance to other drugs in HCC is also summarized (**Figure 3B** **).** We hope to provide a comprehensive understanding of the role of circRNA in drug resistance meanwhile providing potential molecular targets for treatment of drug-resistant HCC.

### 4.5 Roles of circRNA in metabolism

Due to the vast energy demands of cancer cells, their metabolism is often reprogrammed (84–86). Among metabolic “reprogramming”, glucose has been well studied. Recent studies have revealed that some circRNAs are involved in glucose metabolism in HCC. For instance, circMAT2B is prominently overexpressed in HCC tissues and cell lines, predicting unfavorable prognosis. Moreover, PET/CT and HPLC indicate that high expression of circMAT2B is associated with glycolysis in HCC under hypoxic conditions. Mechanistically, circMAT2B promotes glycolysis by increasing pyruvate kinase M2 (PKM2) and kinesin family member C1 (KIFC1) expression by targeting miR-338-3p (87). Moreover, circRNA can simultaneously regulate the metabolism of glucose and other nutrients. Chen et al. found that circ-PRKCI up-regulation increases glucose as well as lactic acid simultaneously and increases forkhead box K1 (FOXK1) expression levels. But that the situation is reversed when FOXK1 is knocked down. Mechanistically, circ-PRKCI increase glucose and lactic acid levels by targeting miR-1294 and miR-186-5p, which bind directly to FOXK1 (88). However, the mechanism by which circ-PRKCI regulates the metabolism of glucose and lactic acid by regulating FOXK1 remains to be further studied. The underlying mechanism of the effect of increased glucose metabolism on lipid metabolism or vice versa also needs to be further elucidated. In addition to the simultaneous regulation of glucose and other nutrient metabolism, circRNA can regulate glucose metabolism and other tumor phenotypes involved in tumor progression simultaneously through more complex mechanisms. Circ-0091579 increases cancer susceptibility candidate 3 (CASC3) expression by sponging miR-490-5p, and the circ-0091579/miR-490-5p/CASC3 axis promotes glycolysis, invasion, migration, and proliferation in HCC (89). A similar study showed that circ-PRMT5 plays an oncogenic role in HCC by modulating proliferation, migration and glycolysis by targeting the miR-188-5p/hexokinase 2 (HK2) axis (90).

Based on these findings, circRNAs play important roles in glucose metabolism and regulate the carcinogenesis and progression of HCC through a variety of complex mechanisms. Additionally, abnormal metabolism in cancers involves various aspects, such as glycometabolism, lipid metabolism, fatty acid metabolism, glutamine metabolism and serine metabolism. Although the role of circRNAs in glycometabolism has been described, additional studies on liquids, fatty acids and others should be performed.

### 4.6 Roles of circRNA in tumor immunology

Accumulating evidence has shown that immunity has an crucial function in the occurrence of cancers (91, 92). In HCC, the immune system is always compromised or dysregulated. Recent studies have revealed that circRNAs are particularly involved in immune dysregulation and modify the function of immune cells. For example, Shi and colleagues demonstrated that the susceptibility of HCC cells to natural killer (NK) cells is influenced by levels of circ-0007456. Furthermore, circ-0007456 controls the cytotoxicity of NK cells by activating the miR-6852-3p/intercellular adhesion molecule-1 (ICAM-1) axis (93). Moreover, by enhancing UL16 binding protein 1 (ULBP1) expression, circARSP91 modulate NK-cell-driven cytotoxicity against HCC (94). In addition, Zhang et al. found that exosomal circUHRF1 induces NK-cell exhaustion by enhancing TIM-3 expression by suppressing miR-449c-5p, and circUHRF1 decreases NK-cell-derived IFN-γ and TNF-α secretion. All of the above factors may result in anti-PD1 therapy resistance (95). NK cells are considered to be the first line of defense for host immune surveillance and play a vital role in antitumor immunotherapy. The above studies have shown that circRNAs regulate the susceptibility, cytotoxicity, exhaustion and cytokine secretion of NK cells, affect the normal function of NK cells in various aspects, and accelerate the progression of HCC. Additionally, macrophages participate in the inflammatory environment of mutagenesis in the early stage of tumor development. As the tumor progresses malignantly, it stimulates angiogenesis, promotes tumor migration and invasion, and inhibits tumor immunity. CircRNAs can regulate the function of macrophages. For example, circ-0110102 inhibits HCC development and macrophage activation through the miR-580-5p/peroxisome proliferator-activated receptor alpha (PPARα)**/**cyclooxygenase-2 (CCL2) signaling pathway (96), hsa_circ_0003410 enhances the proportion of M2/M1 macrophages through the miR-139-3p/CCL5 pathway and facilitates HCC progression (97). By regulating the function of immune cells, circRNAs mediate tumor immune surveillance and promote the progression of HCC.

However, contrary to current opinion, circRNAs not only regulate tumor immunology but are also altered by immune cells. Zhang’s study found that exosomal hsa_circ_0004658 activate by recombination signal binding protein for immunoglobulin Kappa J region (RBPJ) up-regulated macrophages and prevent progression of HCC. Mechanistic investigations have demonstrated that hsa_circ_0004658 sponges miR-499b-5p, thereby regulating expression of junctional adhesion molecule 3 (JAM3) (98).

Together, circRNAs participate in tumor immunology by affecting the functions of immune cells, such as NK cells and macrophages. Moreover, circRNAs are modulated by immune cells, which suggests a complicated relationship between circRNAs and tumor immunology. Due to the roles of circRNAs in tumor immunology and the promising performance of immune checkpoint inhibitors, circRNAs represent potential immune therapeutic targets for HCC.

### 4.7 CircRNA interplay with epigenetic modification

**Figure 4:**
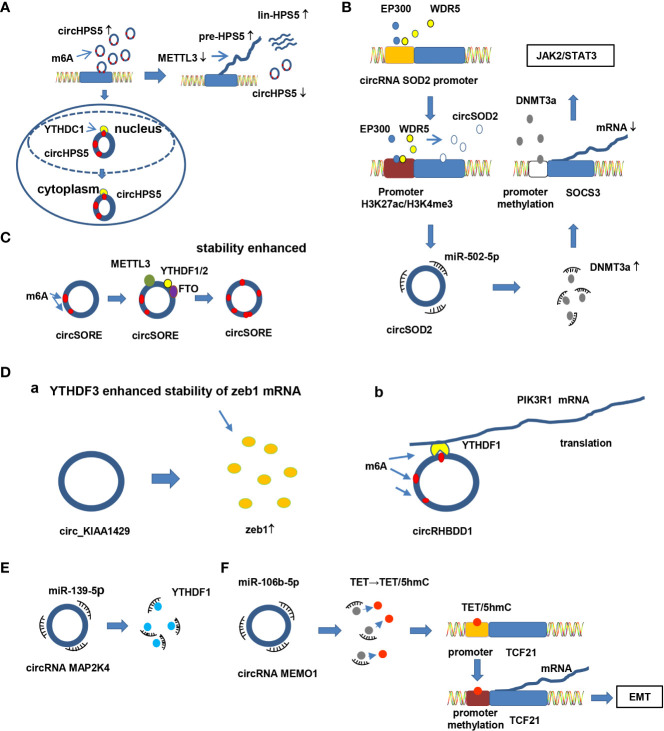
Crosstalk between circRNA and epigenetic modification in HCC. **(A)** Epigenetic modification mediates circRNA splicing and export. CircHPS5 is overexpressed in neoplastic HCC tissues and cells and promotes EMT and CSCs in HCC. Knockdown of METTL3 apparently suppresses circHPS5 but enhances pre-HPS5 and lin-HPS5 expression. Moreover, YTHDC1 enhances the cytoplasmic output of circHPS5 by binding to m6A-modified sites of circHPS5. Knocking down YTHDC1 increases the amount of nuclear circHPS5. **(B)** Proteins bind to the promoter region of circRNAs, promoting expression of circRNAs by modifying epigenetic modification of the promoter. For example, EP300 and WDR5 bind to the promoter region of circRNA SOD2, inducing epigenetic modification (H3K27ac and H3K4me3). The modified promoter increases circRNA SOD2 expression. Overexpression of circSOD2 modulates progression of HCC by suppressing miR-502-5p and activating DNMT3a. Moreover, activated DNMT3a inhibits SOCS3 expression by triggering DNA methylation of the SOCS3 promoter and activating the JAK2/STAT3 pathway. **(C)** m6A enzymes directly bind to circRNAs and increase the stability of circRNAs by increasing m6A levels in circRNAs. For example, METTL3, FTO and YTHDF1/2 directly bind to circSORE and increase its stability by elevating the level of m6A at a specific adenosine. **(D)** CircRNAs interacting with proteins and m6A modification impact interaction between circRNA and proteins. For example, a) circ_KIAA1429 enhances zeb1 expression levels, and YTHDF3 improves the stability of Zeb1 mRNA in an m6A-dependent manner; b) YTHDF1 binds to the m6A modification site of circRHBDD1; YTHDF1 also binds to PIK3R1 mRNA in an “m6A”-dependent manner to promote PIK3R1 expression, thereby facilitating HCC progression. **(E)** CircRNAs regulate expression of enzymes involved in m6A by targeting miRNAs. For example, circRNA MAP2K4 regulates expression of YTHDF1 by sponging miR-139-5p. **(F)** CircRNAs modulate the epigenetic modification of target proteins by sponging miRNAs. For instance, circRNA MEMO1 promotes 5hmC of TET by sponging miR-106b-5p. However, 5hmC of TET facilitates the EMT process by inducing promoter methylation of TCF21, promoting expression of TCF21.

Emerging evidence has verified that epigenetic dysregulation contributes to the initiation and progression of cancer (99–101). Among these epigenetic dysregulations, N6-methyladenosine (m6A) and 5-methylcytosine (m5C) have been well studied (102, 103). Interestingly, some circRNAs have also been demonstrated to participate in HCC tumorigenesis and development **(** **Figure 4** **)**. In general, crosstalk and interplay between circRNA and epigenetic modification are involved in HCC progression, though the details require further elucidation.

In HCC, epigenetic modification can modulate circRNA splicing, export, expression, stability and interaction with proteins, affecting progression of HCC. First, m6A enzymes mediate alternative splicing and export of circRNA thereby affecting the generation, position and function of circRNA. For example, circHPS5 is up-regulated in neoplastic HCC tissues and cells and promotes EMT and cancer stem cells (CSCs) in HCC. CircHPS5 is distributed in both the nucleus and cytoplasm. Further analysis revealed that circHPS5 is highly m6A modified. Knockdown of methyltransferase-like 3 (METTL3), a component of methyltransferases, obviously suppressed circHPS5 expression but enhanced pre-HPS5 and lin-HPS5 expression, suggesting that METTL3 mediates alternative splicing of circHPS5. Moreover, YTH domain-containing 1 (YTHDC1), a “reader” of m6A, increases the cytoplasmic output of circHPS5 by binding to m6A-modified sites of circHPS5. Knockdown of YTHDC1 increases the amount of nuclear circHPS5 (104) **(** **Figure 4A** **)**. Another study showed similar results. Hsa_circ_0058493 promotes the growth and metastasis of HCC cells, whereas knockdown of hsa_circ_0058493 has the opposite effect. In detail, METTL3 regulates the degree of m6A modification of hsa_circ_0058493 and impacts its biological function in HCC. YTHDC1 facilitates localization of hsa_circ_0058493 from the nucleus to the cytoplasm (105). Thus, it can be inferred that METTL3 mediates alternative splicing of circRNA, while YTHDC1 mediates the export of circRNA. M6A enzymes mediate alternative splicing, location and biological functions of circRNA, thereby definitely affecting the tumorigenesis of HCC. Second, epigenetic modification in circRNA promoter regions modulates circRNA expression. As E1A binding protein P300 (EP300) and WD repeat domain 5 (WDR5) bind to the promoter region of circSOD2, the promoter is modified by H3K27ac and H3K4me3, resulting in up-regulation of circSOD2. Subsequently, overexpressed circSOD2 suppresses miR-502-5p expression, activating DNMT3a expression. Activated DNMT3a inhibits SOCS3 expression by triggering DNA methylation of the suppressor of cytokine signaling 3 (SOCS3) promoter, inducing JAK2/STAT3 pathway activity. In contrast, silencing circSOD2 inhibits proliferation, migration and the cell cycle in HCC (106) **(** **Figure 4B** **)**. Third, m6A participates in maintaining the stability of circRNA. For example, the stability of circ-SORE is enhanced with increasing levels of m6A at a specific adenosine in itself. Subsequently, up-regulated circ-SORE activates Wnt/β-catenin signaling by competitively binding to miR-130a-2-5p and miR-660-3p and mediates sorafenib resistance in HCC (71) (**Figure 4C**). Another similar study revealed that insulin like growth factor 2 mRNA binding protein 1 (IGF2BP1), a “reader” of m6A, enhances the stability of circMDK by directly binding to it. Therefore, up-regulated circMDK promotes proliferation, migration and invasion and suppresses apoptosis in HCC *via* the circMDK/miR-346 and miR-874-3p/autophagy related 16 like 1(ATG16L1)/PI3K/AKT/mTOR pathways (107). Fourth, circRNAs interact with proteins, with m6A modification impacting this interaction. For instance, circ-0084922 (circ-KIAA1429) acts as an oncogene in HCC tissues and cells. Overexpression of circ-0084922 facilitates invasion, migration and EMT in HCC by up-regulating Zeb1. Moreover, YTH domain family 3 (YTHDF3), a m6A “reader” enzyme, improves the stability of Zeb1 mRNA in a m6A-dependent manner (108) (**Figure 4Da**). This study provides new insights into the interaction between circRNAs and proteins. However, there are still limitations. In this study, researchers illustrated that YTHDF3 improves the stability of Zeb1 but did not clarify the relationship between YTHDF3 and circ-0084922. Another study complements the understanding of this issue. YTH domain family 1 (YTHDF1), another m6A “reader”, directly binds to circRHBDD1 by acting as a circRHBDD1-interaction protein and subsequently binds to phosphoinositide-3-kinase regulatory subunit 1 (PIK3R1) mRNA, promoting translation of PIK3R1 and regulating metabolic rewiring and anti-PD1 therapy in HCC (109) **(** **Figure 4Db**).

In conclusion, epigenetic modification modulates circRNA alternative splicing, expression and stability by regulating the epigenetic modification status of circRNA itself or the promoter region of circRNA. In addition, the output of circRNA is regulated by identifying the epigenetic modification sites in circRNA *via* m6A “readers”.

Epigenetic modification can not only modulate circRNA alternative splicing, expression, export, stability and interaction with proteins but also be regulated by circRNAs, promoting or suppressing carcinogenesis, growth and progression of HCC. In HCC, circRNAs regulate the expression of m6A-related enzymes or the epigenetic modification status of proteins *via* the interplay among circRNA-miRNA interactions, thereby modifying their biological behavior.

For example, YTHDF1 is a component of the m6A “reader” and plays an essential role in HCC development. Chi et al. demonstrated that circMAP2K4 mediates the proliferation of HCC by governing expression of YTHDF1 by sponging miR-139-5p (110) (**Figure 4E**). In addition, circMEMO1 induces TET family high 5-hydroxymethylcytosine (5hmC) levels by sponging miR-106b-5p, which further modulates methylation of the transcription factor 21 (TCF21) promoter region. High promoter methylation promotes TCF21 expression, resulting in EMT activation (111) (**Figure 4F**).

In conclusion, circRNA can regulate the progression of epigenetic modification and be modulated by epigenetic modifications, indicating a complex interplay between circRNA and epigenetic modification. These findings concerning epigenetic modification and circRNAs provide new insight into the pathogenesis of HCC and may allow for additional breakthroughs in HCC treatment.

## 5 Novel implications of circRNA in HCC

### 5.1 CircRNAs as diagnostic and prognostic biomarkers

Of note, with the rapid development of high-throughput sequencing technologies and bioinformatics, a multitude of noncoding RNAs have been identified. Emerging evidence has indicated that noncoding RNAs such as microRNAs, lncRNAs, and circRNAs, as well as some proteins, have the potential to serve as diagnostic and prognostic biomarkers for HCC. As covalently closed loop structures, circRNAs display increased stability against RNase compared to linear RNAs, such as microRNAs, lncRNAs and mRNAs (5, 51), and are more quantifiable than proteins by RT−qPCR and droplet digital PCR (ddPCR). In addition, circRNAs are widely found in blood, saliva and other body fluids (112–114), which may provide a noninvasive method for detection; circRNAs are also tissue and stage specific, increasing the sensitivity and specificity of HCC diagnosis. Therefore, we believe that circRNAs are superior to other potential diagnostic and prognostic biomarkers for HCC. Furthermore, accumulating evidence has confirmed the probable role of circRNAs as diagnostic biomarkers for HCC.

To date, several studies have reported potential diagnostic and prognostic value in HCC for single circRNAs. For example, Weng et al. found circ-0064428 expression to be apparently decreased in high tumor-infiltrating lymphocyte (TIL) patients compared to low TIL patients, and it was associated with the survival, tumor size and metastasis of HCC patients, constituting a potential HCC prognostic biomarker. Kaplan−Meier survival curves revealed that the difference in overall survival (OS) between the circ-0064428 high and low expression groups (24 months was 0.36 vs. 0.72; 60 months was 0.16 vs. 0.27) was statistically significant. In tumors larger than 5 cm, high and low circ_0064428 expression was 58.3% and 26.7%, respectively. In addition, high expression of hsa_circ_0064428 was more likely to develop into advanced tumor stage III (56.7% vs. 20.0%, *p*<0.01) and lead to metastasis (20.0% vs. 10.0%) (115). Another study conducted by Lei et al. showed that 58 circRNAs are dysregulated in peripheral blood mononuclear cells of HCC patients compared to healthy donors. Among the six chosen circRNAs, circ-0000798 was found to correlate with clinical features. Furthermore, overexpression of circ-0000798 correlated with poor OS in HCC patients and helped to distinguish them from healthy controls. The area under the curve (AUC) of circ_0000798 was 0.703, suggesting that is has the capacity to act as a tumor marker (113). Moreover, circ_0004913 was identified as a potential biomarker for HCC monitoring and prognosis. Li’s study investigated 150 HCC patient tissues after surgery and found circ_0004913 and circ_0008160 to be down-regulated but circ_0000517 up-regulated in HCC tissue. Further studies found that increased expression of circ_0004913 is negatively related to largest tumor size and correlated with longer OS and that it might act as a monitoring and prognostic biomarker for HCC (116). Therefore, a single circRNA has been demonstrated to have excellent performance in HCC diagnosis and prognosis, yet the combination of several circRNAs performs significantly better. Circ-0004001, circ-0004123, and circ-0075792 are up-regulated in HCC blood samples, which correlated positively with TNM stage and tumor size. Alone or in combination, these three circRNAs exhibited good sensitivity and specificity for the diagnosis of HCC. When used separately as a diagnostic marker, the AUC, cut-off, sensitivity and specificity of circ-0004001 were 0.79, 51.43, 76.19%, and 81.25%, the circ-0004123 values were 0.73, 221.7, 66.67%, and 84.38%, and the circ-0075792 values were 0.76, 79.48, 80.95%, and 68.75%, respectively, while they increased to 0.89, 0.33, 90.5%, and 78.1% when combined. Their combination obviously improved the AUC, cut-off and sensitivity of HCC diagnosis; however, for specificity, the combination showed no significant change. Overall, their combination is preferable to the individual used (117). This study verified the potential value of circRNA combinations for the diagnosis of HCC.

Additionally, serum alpha-fetoprotein (AFP) has correlated been used for detecting HCC, with obvious low sensitivity and specificity. Compared with AFP, circRNA shows great superiority with regard to sensitivity and specificity of HCC diagnosis. Compared to AFP, Yu and colleagues reported increased accuracy of the circPanel in distinguishing HCC from chronic hepatitis B (CHB), HBV-related liver cirrhosis and heathy controls; it also exhibited improved accuracy in differentiating among small HCC, AFP-negative HCC and AFP-negative small HCC from HCC.In this study, Yu and colleagues collected plasma, HCC and adjacent noncancerous liver (ANL) tissues from three different hospitals, and each hospital was divided into a set. Each set was further divided into four subgroups (HCC vs. non-HCC/healthy/CHB/cirrhosis) for further study. In the diagnosis of HCC, the AUC of circPanel ranged from 0.858 to 0.87, which was significantly higher than that of AFP (0.76 to 0.84) in the four subgroups of the training set. Similarly, in the diagnosis of small HCC, the AUC of circPanel was approximately 0.86 (0.857 to 0.869), while the AUC of AFP in the four subgroups ranged from 0.65 to 0.731, which was significantly lower than that of circPanel. Moreover, the circPanel also had a high diagnostic accuracy (all AUCs were higher than 0.800) in the diagnosis of AFP-negative HCC and AFP-negative small HCC. The other set showed a similar trend, confirming the results (118).

In addition, circRNAs combined with AFP may be more sensitive and specific for HCC diagnosis and prognosis. Wu and colleagues determined that circulating circ-0009582, circ-0037120 and circ-0140117 are able to discriminate HCC of HBV-positive chronic hepatitis or negative control, and the three circRNAs exhibited better sensitivity and specificity when combined with AFP. In the training set, the AUCs of circ_0009582, circ_0037120, and circ_0140117, the combination of circRNAs (factors), AFP, and the combination of circRNAs and AFP (merged) were 0.688, 0.742, and 0.762, 0.800, 0.740 and 0.988, respectively. The values in the validation set were 0.805, 0.835, 0.845, 0.857, 0.803 and 0.955 (119). Liu’s study found that circ-0005397 up-regulation is associated with tumour size and TNM stage in HCC and predicts poor OS. Dynamic monitoring of circ-0005397 might be useful for predicting recurrence and metastasis of HCC after surgery. Compared with circ-0005397(82.0%), circ-0005397+AFP (89.9%) and circ-0005397+AFP-L3 (87.6%), the combination of circ-0005397, AFP and AFP-L3 enhanced the sensitivity (93%) of HCC diagnosis (114).

Together, we infer that circRNAs are easily detected in body fluid samples and are relevant to the clinical manifestation and prognosis of HCC. When used alone or in combination with other circRNAs or AFP, circRNAs show higher accuracy in distinguishing HCC from benign lesions and heathy controls. In conclusion, circRNAs are promising biomarkers for HCC diagnosis and prognosis. Although circRNAs have potential as biomarkers for HCC, there are still many questions for their clinical application. For instance, in addition to AFP, other tumor biomarkers, such as CA125, CA199, and CA724, are used for HCC diagnosis. However, there are few studies on circRNAs in combination with other tumor biomarkers. Additionally, it remains unknown whether “core” circRNAs exist. Moreover, large, multicenter randomized trials are necessary to determine the true efficacy of circRNAs in detecting HCC in healthy people. More research is needed to answer these questions.

### 5.2 CircRNAs as potential therapeutic targets

Despite aggressive treatment, the mortality of HCC is still high. Frequent relapses and metastasis are the main causes of HCC death. Therefore, effective therapeutic targets and new therapeutic strategies are urgently needed. Accumulating evidence has demonstrated that knockdown or exogenous overexpression of circRNA regulates the tumorigenesis and progression of HCC, suggesting that circRNAs represent therapeutic targets (**Table 2**). Circ-0001649 is downregulated in HCC tissues, and gain of circ-0001649 function suppresses the progression of HCC, attenuating proliferation, migration, invasion and activating apoptosis (121). Conversely, circ-0085616 is overexpressed in HCC cells, and the proliferative capacity of HCC is restrained by decreasing circ-0085616, demonstrating that circ-0085616 might be a novel therapeutic target for HCC (122). Furthermore, Zhang et al. found that knockdown of circ-0008450 restrains proliferation, invasion and migration in Huh-7 cells as well as promotes apoptosis. In contrast, overexpression of circ-0008450 induces the opposite results in HepG2 cells (120). *In vivo* and *in vitro* experiments have indicated that overexpression of circDLC1 decreases cell viability and motility in HCC through the circDLC1/HuR/matrix metallopeptidase 1 (MMP1) axis. Silencing circDLC1 reverses these results (15). Collectively, these results suggest that circRNAs may serve as therapeutic targets for HCC.

**Table 2:** Expression, functional characterization and clinical application of circRNAs in HCC.

CircRNAs	Expression	Mechanism	Target genes and signalling pathway	Clinical application	References
circDLC1	↓	binding to RNA-binding protein	CircDLC1/HuR/MMP1	prognostic marker; therapeutic target	(15)
circSEC24A(hsa_circ_0003528)	↑	sponging miRNA	CircSEC24A/miR-421/MMP3	therapeutic target	(64)
circFGGY(hsa_circ_0006633)	↓	sponging miRNA	CircFGGY/miR-545-3p/Smad7	prognostic biomarker	(65)
circ_0008450	↑	sponging miRNA	circ_0008450/miR‐548p	therapeutic target	(120)
hsa_circ_0004658	↑	sponging miRNA	hsa_circ_0004658/miR-499b-5p/JAM3	diagnostic biomarker; therapeutic target	(98)
circHPS5	↑	sponging miRNA	CircHPS5/miR-370/HMGA2	Prognostic biomarker therapeutic target	(104)
hsa_circ_0058493	↑	bingding protein	METTL3/hsa_circ_0058493/YTHDC1	therapeutic target	(105)
circMDK	↑	sponging miRNA	circMDK/miR-346 and miR-874-3p/ATG16L1/PI3K/AKT/mTOR pathway	nanotherapeutic target	(107)
circMAP2K4	↑	sponging miRNA	CircMAP2K4/miR-139-5p/ YTHDF1	therapeutic target	(110)
circ_0004913	↓			Prognostic biomarker	(116)
hsa_circ_0004001, hsa_circ_0004123, hsa_circ_0075792	↑	targeting miRNA	participated in VEGF/ VEGFR, PI3K/Akt, mTOR, and Wnt signalling pathways	diagnosis alone or combination	(117, 120)
hsa_circ_0001649	↓			prognostic biomarker; therapeutic target	(121)
hsa_circ_0085616	↑		hsa_circ_0085616/β-catenin, p-ERK, and p-AKT	biomarker; therapeutic target	(122)
circRNA cSMARCA5 (hsa_circ_0001445)	↓	sponging miRNA	cSMARCA5/miR-17–3p or miR-181b-5p/TIMP3.	therapeutic target	(123)
circRHOT1 (hsa_circRNA_102034)	↑	circRHOT1 recruited TIP60 to the NR2F6 promoter and initiated NR2F6 transcription	NR2F6	prognosis biomarkers	(124)
circ-CDYL	↑	sponging miRNA	Circ-CDYL/miR-892a/HDGF/NCL/PI3K-AKT;Circ-CDYL/miR-328–3p/HIF1AN/NOTCH2	diagnosis alone or combined with HDGF and HIF1AN	(125)
circASAP1	↑	competing endogenous RNA(ceRNA)	CircASAP1/miR-326 or miR-532–5p/MAPK1 and CSF-1	prognostic predictor	(126)
circRNA_104075	↑	absorbing miRNA	HNF4a/circRNA_104075/miR-582–3p/YAP	diagnostic biomarker	(127)
circ-ADD3	↓	regulation of EZH2 stability	circ-ADD3/EZH2	biomarker for diagnosis and prognosis	(128)
hsa_circ_00156, hsa_circ _000224 and hsa_circ_000520	circ _000224↑ circ_000520↓ circ_00156↓			diagnosis biomarker alone or combination	(129)
hsa_circ_0000517	↑		hsa_circ_0000517/miRNA/MAPK and Ras pathway; hsa_circ_0000517/TP53, MYC, and AKT1.	prognosis biomarker	(130)
hsa_circ_0028502 hsa_circ_0076251	↓			diagnosis biomarkers prognostic indicator	(131)
hsa_circ_0003998	↑		alone or combination with AFP	diagnosis and prognosis biomarker	(132)
hsa_circ_0128298	↑			diagnostic and prognostic biomarker	(133)
circRNA SMARCA5	↓			prediction and monitor biomarker	(134)
hsa_circ_0001445	↓			diagnostic biomarker	(135)
hsa_circ_0078602	↓			diagnostic biomarker	(136)
circRNA_101237	↑			prognostic biomarker; therapeutic target	(137)
CircMAP3K4	↑	Encode peptide	CircMAP3K4/IGF2BP1/circMAP3K4-455aa/AIF or MIB1	prognostic factor	(138)
CircMTO1	↓	sponging miRNA	CircMTO1/miR-541-5p/ZIC1/Wnt/β-catenin	therapeutic target	(139)

Symbols ↑ represents “Up”, and symbols ↓ represents “Down”.

Although circRNAs have the potential to be therapeutic targets for HCC, appropriate treatment strategies are also urgently needed. Recently, several therapeutic strategies have been reported to target circRNAs for HCC treatments, broadening the horizons of circRNAs as “druggable” targets. Common strategies for knocking down or knocking out circRNAs include strategies based on RNA interference (RNAi) - siRNA and shRNA, strategies based on the clustered regularly interspaced short palindromic repeats (CRISPR)/CRISPR-associated protein 9 (Cas9), strategies based on CRISPR/Cas13 and other strategies, such as the cre-lox system. RNAi-based strategies are the most mature and convenient method for knocking down circRNAs *in vivo* and are widely used in the study of circRNAs in HCC (140–142). Compared with RNAi technology, the CRISPR/Cas9-based strategy showed high specificity and efficiency in genome editing of circRNAs knockout. Moreover, the CRISPR/Cas9 system successfully knocked out circRNAs *in vivo* and *in vitro*. In HCC, Gu and colleagues employed CRISPR/Cas9 technology to generate circIPO11 knockout mice (143). Zhao and colleagues used CRISPR/Cas9 to silence circSOD2 *in vitro* (106). However, few studies on circRNAs in HCC have used the CRISPR/Cas13 system and the cre-lox system. In addition to circRNA knockout or knockdown, exogenous overexpression is a key means to study the biological function of circRNA. Plasmid (142), lentiviral (141) vectors are often used for circRNA overexpression. Currently, overexpression of circRNAs by direct synthesis and purification provides a new idea for exogenous overexpression of circRNA. In addition, nanoparticle and exosome delivery systems are used for circRNA-based therapeutics, encapsulating siRNA and circRNA expressing vectors into cells (95, 107, 144). Although there are many therapeutic strategies for HCC *via* targeting circRNAs, there are still many obstacles in the process of achieving these methods, such as off-target gene silencing (145, 146), mis-spliced products (147), and triggering an immune response (148). Therefore, there is still a long way to go for these therapeutic strategies to be truly applied in clinical practice.

### 5.3 New drugs targeting circRNAs

It can be inferred from the above that circRNAs play decisive roles in the carcinogenesis and development of HCC, and gain or loss of their function may disrupt the initiation and progression of HCC. To date, numerous drugs have been demonstrated to exert antitumor activities against HCC by targeting circRNAs (**Table 3** **)**. For instance, circ-100338 was found to be markedly increased in HCC cell lines. Overexpression of circ-100338 promotes the proliferation of HCC through the miR-141-3p/ZEB1 axis. However, piplartine decreases expression of circ-100338, resulting in attenuated HCC proliferation (149). Si et al. demonstrated that celastrol has an antitumor effect on HCC by down-regulating circ-SLIT3. Mechanistically, less circ-SLIT3 binds directly to miR-223-3p, suppressing C-X-C motif chemokine receptor 4 (CXCR4) expression (150). In addition, matrine was demonstrated to suppress the development of HCC by inhibiting cell growth, migration and invasion while facilitating apoptosis and autophagy. Circ_0027345 is down-regulated by matrine, but exogenous circ_0027345 counters the effect of matrine on cell progression. In detail, matrine suppresses HCC development *via* the circ-0027345/miR-345-5p/homeobox D3 (HOXD3) axis (151). Furthermore, androgen receptor inhibits metastasis and invasion and formation of vasculogenic mimicry (VM) in HCC. Androgen receptor suppresses VM by directly targeting host gene promoter regions of circR7, subsequently inhibiting expression of circR7. Down-regulated circR7 activates miR-7-5p/VE-cadherin/Notch4 signaling and impedes VM formation (152). In conclusion, modern drugs restrain the progression of HCC by targeting circRNAs. Despite the excellent antitumor effects that new drugs have shown, the mechanisms of novel drug anti-HCC need to be identified. Moreover, additional large-scale clinical studies must be performed to implement circRNAs as therapeutic targets for clinical treatment.

**Table 3:** Summary of drugs and their targeted circRNAs in HCC.

Drug name	CircRNA	Dysregulation in HCC	Functions of drugs	Signalling pathway	Reference
Piplartine	circ-100338	↑	Inhibiting proliferation	circ-100338/miR- 141-3p/ZEB1 axis	(149)
Celastrol	circ-SLIT3	↑	Inhibiting proliferation, migration, invasion, and enhanced apoptosis	circ-SLIT3/miR-223-3p/CXCR4	(150)
Matrine	Circ_0027345	↑	Inhibiting cell growth, migration and invasion, facilitating apoptosis and autophagy	circ-0027345/miR- 345-5p/HOXD3 axis	(151)
Androgen receptor	circR7	↑	Inhibits metastasis and invasion and formation of VM	circR7/miR-7-5p/ VE-cadherin/Notch4 signalling	(152)

Symbols ↑ represents “Up”.

## 6 Perspectives and conclusions

become a hot research topic in tumor biology and therapy. A great number of studies have demonstrated that circRNAs are obviously dysregulated in HCC cells, tissues and body fluids. Some circRNAs have been proven to modulate the initiation, growth and progression, such as proliferation, migration and invasion, apoptosis, drug resistance, metabolism, tumor immunity, and epigenetic progression of HCC. Additionally, circRNAs are promising as diagnostic and prognostic biomarkers or effective therapeutic targets for HCC.

Although circRNAs have exerted important roles in HCC, there are still many obstacles in circRNA research. The nomenclature of circRNA is a fundamental issue in the field of circRNA research. However, the nomenclature of circRNAs is quite ambiguous (14). Since the confusing naming of circRNAs has caused many problems for subsequent studies, a more scientific naming method is urgently needed. Recently, some professional websites, such as circBank (17), have been working to create a more standardized naming system for circRNAs, but there is still a long way to go before the new naming system is recognized and widely used, which indicates the difficulty of studying circRNA for understanding HCC development or therapy or diagnosis.

Although some recent studies on the biological function and clinical application of circRNAs have been carried out, laboratory and clinical studies of HCC are lacking. For example, tumor drug resistance is a common issue in HCC treatment. According to 2021 NCCN Guidelines for Hepatobiliary Cancers, atezolizumab+bevacizumab (A+T) and oxaliplatin + calcifolinate + 5-fluorouracil (FOLFOX) are recommended as first-line treatments. Regorafenib, cabozantinib, ramucirumab, ipilimumab and pembrolizumab are recommended as subsequent-line treatments (68); sorafenib, lenvatinib and nivolumab are recommended as first-line and subsequent-line treatments. However, due to laging far behind clinical practice, recent studies on drug resistance have primarily focused on sorafenib (70–73), programmed death 1 (PD1) (76, 77) and oxaliplatin (78), with scarce related research on lenvatinib, atezolizumab, bevacizumab and other drugs. Therefore, many unknowns on resistance to recommended drugs remain, and there are few methods for intervening in drug resistance. Thus, additional effort and resources should be applied to study drug resistance mediated by circRNAs. Furthermore, epigenetic modification is a hot area of research, and the interplay between epigenetic modification and circRNA has attracted much attention. Nevertheless, research on HCC in this field is still insufficient. In a study of the influence of m6A modification on circRNA metabolism in HeLa cells, Timoteo and colleagues revealed that different mutation sites control circ-ZNF609 formation and METTL3-YTHDC1-mediated back-splicing of circRNA. Moreover, m6A modification modulates circ-ZNF609 translation *via* YTHDF3 and eIF4G2 (153). However, there have been few high-quality studies in HCC. Accordingly, a high-quality and in-depth study should be carried out to explore the relationship between epigenetic modification and circRNA in HCC. Additionally, circRNAs have been proven to be promising biomarkers and therapeutic targets in HCC. However, the number of confirmed cases is large, and whether “core” circRNA exists is still unknown. More research should be conducted to address this issue.

Although an increasing number of circRNAs have been identified in HCC through high-throughput sequencing technologies and bioinformatics, only a small number of circRNAs have been studied. Research conducted by Qiu found a total of 92204 circRNAs were involved in HCC tumor and matched peritumor tissues. Of these circRNAs, 20404 (23.13%) have been identified in other studies reported in circBase (154), and the rest (77.87%) are novel (67). Hence, most of the functions and mechanisms of circRNA in HCC are still unknown. Additionally, although some circRNAs have been well studied, our understanding of their function and mechanism is incomplete. For example, hsa_circ_0008450 was found to be up-regulated in hypoxia-exposed HCC cells and HCC tissues, promoting cell growth and glycolysis while suppressing apoptosis of HCC *via* the miR-431/A-kinase anchor protein 1 (AKAP1) axis (155). Another study revealed that high expression of hsa_circ_0008450 correlates with poor prognosis and facilitates cell viability, migration and invasion and inhibits apoptosis by targeting miR-548p (120). Lin et al. indicated that hsa_circ_0008450 promotes HCC progression by modulating the miR-214-3p/EZH2 axis (156). Although a number of studies have been conducted to elucidate the role of hsa_circ_0008450 in HCC, the potential function of hsa_circ_0008450 in autophagy, drug resistance, and tumor immunity, among others is still unknown. In addition, circRNAs are involved in cellular processes in a variety of ways, such as sponging microRNAs, interacting with RBPs, regulating alternative splicing, parental gene transcription, encoding peptides and decoying proteins (157). However, studies to date on circRNAs have mostly focused on miRNA sponges. In addition to miRNAs, ncRNAs also contain other different types, such as lncRNAs, piRNAs, and tsRNAs, and there is little crosstalk between circRNAs and other ncRNAs. Therefore, research on circRNAs in HCC is still in its infancy. Researchers should reveal multiple mechanisms of circRNAs involved in HCC progression.

In addition, some researchers have recently proposed employing exosomes to package circRNAs for HCC treatment, as exosomes are good targeted drug delivery tools and the dose of circRNAs in exosomes is measurable (158). Of course, exosomal circRNAs are a good choice for cancer treatment. Further steps should be taken to make this a reality. Collectively, circRNAs play a vital role in hepatocarcinogenesis and development, serving as potential biomarkers and therapeutic targets for HCC.

## Author contributions

ZL drafted, edited and revised the manuscript. FY and ZX drafted the images and graphs. YL collected and analyzed the published studies. All authors contributed to the article and approved the submitted version.

## Glossary

**Table:**

HCC	hepatocellular carcinoma
circRNAs	circular RNAs
lncRNAs	long non-coding RNAs
EMT	epithelial-mesenchymal transition
RBPs	RNA-binding proteins
ecircRNAs	exonic circRNAs
HASMCs	human aortic smooth muscle cells
Gata4	GATA-binding protein 4
NPM1	nucleophosmin 1
EIciRNAs	exon‒intron circRNAs
ciRNAs	circular intronic RNAs
tricRNAs	tRNA intronic circRNAs
miRNAs	microRNAs
RNase	ribonuclease
MAPK1	mitogen-activated protein kinase 1
MAPK14	mitogen-activated protein kinase 14
GRAMD1A	GRAM domain-containing protein 1A
Cul2	cullin2
PD-L1	programmed death-ligand 1
PCBP1	poly (rC) binding protein 1
ceRNA	competing endogenous RNA
FOSL2	FOS-like antigen 2
LAMC2	laminin subunit gamma 2
TME	tumor microenvironment
RTKN2	rhotekin-2
ADAMTS13	ADAM metallopeptidase with thrombospondin type 1 motif 13
FDA	US Food and Drug Administration
E2F1	E2F transcription factor 1
MECP2	methyl-CpG binding protein 2
RELN	reelin
LDHA	lactate dehydrogenase A
TKI	tyrosine kinase inhibitor
USP28	ubiquitin-specific protease 28
FOLFOX	oxaliplatin + 5-fluorouracil + calcium folinate
ABCB1	ATP binding cassette transporter subfamily B member 1
DDP	cisplatin
POU3F2	POU class 3 homeobox 2
NOR1	oxidored-nitro domain-containing protein 1
ABCC1	ATP binding cassette subfamily C member 1
PD1	programmed death 1
DPP4	dipeptidyl peptidase 4
PKM2	pyruvate kinase M2
KIFC1	kinesin family member C1
FOXK1	forkhead box K1
CASC3	cancer susceptibility candidate 3
HK2	hexokinase 2
NK	natural killer
ICAM-1	intercellular adhesion molecule-1
ULBP1	UL16 binding protein 1
PPARα	peroxisome proliferator-activated receptor alpha
CCL2	cyclooxygenase-2
RBPJ	recombination signal binding protein for immunoglobulin Kappa J region
JAM3	junctional adhesion molecule 3
m6A	N6-methyladenosine
m5C	5-methylcytosine
CSCs	cancer stem cells
METTL3	methyltransferase-like 3
YTHDC1	YTH domain-containing 1
EP300	E1A binding protein P300
WDR5	WD repeat domain 5
SOCS3	suppressor of cytokine signaling 3
IGF2BP1	insulin like growth factor 2 mRNA binding protein 1
ATG16L1	autophagy related 16 like 1
YTHDF3	YTH domain family 3
YTHDF1	YTH domain family 1
PIK3R1	phosphoinositide-3- kinase regulatory subunit 1
5hmC	5-hydroxymethylcytosine
TCF21	transcription factor 21
ddPCR	droplet digital PCR
TIL	tumor-infiltrating lymphocyte
OS	overall survival
AUC	area under the curve
AFP	alpha-fetoprotein
CHB	chronic hepatitis B
ANL	adjacent noncancerous liver
MMP1	matrix metallopeptidase 1
CRISPR	clustered regularly interspaced short palindromic repeats
Cas9	CRISPR-associated protein 9
CXCR4	C-X-C motif chemokine receptor 4
HOXD3	homeobox D3
VM	vasculogenic mimicry
A+T	atezolizumab+bevacizumab
AKAP1	A-kinase anchor protein 1

## References

[1] Global Burden of Disease Liver Cancer Collaboration; Akinyemiju T Abera S Ahmed M Alam N Alemayohu MA. The burden of primary liver cancer and underlying etiologies from 1990 to 2015 at the global, regional, and national level: Results from the global burden of disease study 2015. JAMA Oncol (2017) 3:1683–91. doi: 10.1001/jamaoncol.2017.3055 PMC5824275 28983565

[2] Kanwal F Singal AG. Surveillance for hepatocellular carcinoma: Current best practice and future direction. Gastroenterology (2019) 157:54–64. doi: 10.1053/j.gastro.2019.02.049 30986389 PMC6636644

[3] Sanger HL Klotz G Riesner D Gross HJ Kleinschmidt AK. Viroids are single-stranded covalently closed circular RNA molecules existing as highly base-paired rod-like structures. Proc Natl Acad Sci USA (1976) 73:3852–6. doi: 10.1073/pnas.73.11.3852 PMC431239 1069269

[4] Jeck WR Sharpless NE. Detecting and characterizing circular RNAs. Nat Biotechnol (2014) 32:453–61. doi: 10.1038/nbt.2890 PMC4121655 24811520

[5] Jeck WR Sorrentino JA Wang K Slevin MK Burd CE Liu J. Circular RNAs are abundant, conserved,and associated with ALU repeats. RNA (2013) 19:141–57. doi: 10.1261/rna.035667.112 PMC3543092 23249747

[6] Salzman J Chen RE Olsen MN Wang PL Brown PO. Cell-type specific features of circular RNA expression. PLoS Genet (2013) 9:e1003777. doi: 10.1371/journal.pgen.1003777 24039610 PMC3764148

[7] Nicolet BP Engels S Aglialoro F van den Akker E von Lindern M Wolkers MC. Circular RNA expression in human hematopoietic cells is widespread and cell-type specific. Nucleic Acids Res (2018) 19:8168–80. doi: 10.1093/nar/gky721 PMC6144802 30124921

[8] Zhang P Zuo Z Shang W Wu A Bi R Wu J. Identification of differentially expressed circular RNAs in human colorectal cancer. Tumour Biol (2017) 39:1010428317694546. doi: 10.1177/1010428317694546 28349836

[9] Westholm JO Miura P Olson S Shenker S Joseph B Sanfilippo P. Genome-wide analysis of drosophila circular RNAs reveals their structural and sequence properties and age-dependent neural accumulation. Cell Rep (2014) 9:1966–80. doi: 10.1016/j.celrep.2014.10.062 PMC4279448 25544350

[10] Liu Z Chen Q Hann SS. The functions and oncogenic roles of CCAT1 in human cancer. BioMed Pharmacother (2019) 115:108943. doi: 10.1016/j.biopha.2019.108943 31078038

[11] Hansen TB Jensen TI Clausen BH Bramsen JB Finsen B Damgaard CK. Natural RNA circles function as efficient microRNA sponges. Nature (2013) 495:384–8. doi: 10.1038/nature11993 23446346

[12] Liu B Yang G Wang X Liu J Lu Z Wang Q. CircBACH1 (hsa_circ_0061395) promotes hepatocellular carcinoma growth by regulating p27 repression *via* HuR. J Cell Physiol (2020) 235:6929–41. doi: 10.1002/jcp.29589 32003018

[13] Zhang XO Wang HB Zhang Y Lu X Chen LL Yang L. Complementary sequence-mediated exon circularization. Cell (2014) 159:134–47. doi: 10.1016/j.cell.2014.09.001 25242744

[14] Vromman M Vandesompele J Volders PJ. Closing the circle: current state and perspectives of circular RNA databases. Brief Bioinform (2021) 22:288–97. doi: 10.1093/bib/bbz175 PMC7820840 31998941

[15] Liu H Lan T Li H Xu L Chen X Liao H. Circular RNA circDLC1 inhibits MMP1-mediated liver cancer progression *via* interaction with HuR. Theranostics (2021) 11:1396–411. doi: 10.7150/thno.53227 PMC7738888 33391541

[16] Xu L Feng X Hao X Wang P Zhang Y Zheng X. CircSETD3 (Hsa_circ_0000567) acts as a sponge for microRNA-421 inhibiting hepatocellular carcinoma growth. J Exp Clin Cancer Res (2019) 38:98. doi: 10.1186/s13046-019-1041-2 30795787 PMC6385474

[17] Liu M Wang Q Shen J Yang BB Ding X. Circbank: a comprehensive database for circRNA with standard nomenclature. RNA Biol (2019) 16:899–905. doi: 10.1080/15476286.2019.1600395 31023147 PMC6546381

[18] Yin L Yao J Deng G Wang X Cai W Shen J. Identification of candidate lncRNAs and circRNAs regulating WNT3/β-catenin signaling in essential hypertension. AGING (2020) 12:8261–88. doi: 10.18632/aging.103137 PMC7244030 32392180

[19] Sekar D. Circular RNA: a new biomarker for different types of hypertension. Hypertens Res (2019) 42:1824–5. doi: 10.1038/s41440-019-0302-y 31316171

[20] Zheng S He X Sun J Li Q Zhang T Zhang L. The up-regulated hsa-circRNA9102-5 may be a risk factor for essential hypertension. J Clin Lab Anal (2020) 34:e23339. doi: 10.1002/jcla.23339 32445294 PMC7439346

[21] Li H Xu JD Fang XH Zhu JN Yang J Pan R. Circular RNA circRNA_000203 aggravates cardiac hypertrophy *via* suppressing miR-26b-5p and miR-140-3p binding to Gata4. Cardiovasc Res (2020) 116:1323–34. doi: 10.1093/cvr/cvz215 PMC7243276 31397837

[22] Jiang Q Liu C Li CP Xu SS Yao MD Ge HM. Circular RNA-ZNF532 regulates diabetes-induced retinal pericyte degeneration and vascular dysfunction. J Clin Invest (2020) 130:3833–47. doi: 10.1172/JCI123353 PMC7324174 32343678

[23] Liu Y Chen S Zong ZH Guan X Zhao Y. CircRNA WHSC1 targets the miR-646/NPM1 pathway to promote the development of endometrial cancer. J Cell Mol Med (2020) 24:6898–907. doi: 10.1111/jcmm.15346 PMC7299690 32378344

[24] Wang J Zhao X Wang Y Ren F Sun D Yan Y. circRNA-002178 act as a ceRNA to promote PDL1/PD1 expression in lung adenocarcinoma. Cell Death Dis (2020) 11:32. doi: 10.1038/s41419-020-2230-9 31949130 PMC6965119

[25] Ni C Yang S Ji Y Duan Y Yang W Yang X. Hsa_circ_0011385 knockdown represses cell proliferation in hepatocellular carcinoma. Cell Death Discovery (2021) 7:270. doi: 10.1038/s41420-021-00664-0 34599150 PMC8486831

[26] Hsu MT Coca-Prados M. Electron microscopic evidence for the circular form of RNA in the cytoplasm of eukaryotic cells. Nature (1979) 280:339–40. doi: 10.1038/280339a0 460409

[27] Cocquerelle C Daubersies P Majérus MA Kerckaert JP Bailleul B. Splicing with inverted order of exons occurs proximal to large introns. EMBO J (1992) 11:1095–8. doi: 10.1002/j.1460-2075.1992.tb05148.x PMC556550 1339341

[28] Cocquerelle C Mascrez B Hétuin D Bailleul B. Mis-splicing yields circular RNA molecules. FASEB J (1993) 7:155–60. doi: 10.1096/fasebj.7.1.7678559 7678559

[29] Nigro JM Cho KR Fearon ER Kern SE Ruppert JM Oliner JD. Scrambled exons. Cell (1991) 64:607–13. doi: 10.1016/0092-8674(91)90244-s 1991322

[30] Pasman Z Been MD Garcia-Blanco MA. Exon circularization in mammalian nuclear extracts. RNA (1996) 2:603–10. PMC1369399 8718689

[31] Conn SJ Pillman KA Toubia J Conn VM Salmanidis M Phillips CA. The RNA binding protein quaking regulates formation of circRNAs. Cell (2015) 160:1125–34. doi: 10.1016/j.cell.2015.02.014 25768908

[32] Errichelli L Modigliani S Laneve P Colantoni A Legnini I Capauto D. FUS affects circular RNA expression in murine embryonic stem cell-derived motor neurons. Nat Commun (2017) 8:14741. doi: 10.1038/ncomms14741 28358055 PMC5379105

[33] Ivanov A Memczak S Wyler E Torti F Porath HT Orejuela MR. Analysis of intron sequences reveals hallmarks of circular RNA biogenesis in animals. Cell Rep (2015) 10:170–7. doi: 10.1016/j.celrep.2014.12.019 25558066

[34] Liang D Wilusz JE. Short intronic repeat sequences facilitate circular RNA production. Genes Dev (2014) 28:2233–47. doi: 10.1101/gad.251926.114 PMC4201285 25281217

[35] Ashwal-Fluss R Meyer M Pamudurti NR Ivanov A Bartok O Hanan M. circRNA biogenesis competes with pre-mRNA splicing. Mol Cell (2014) 56:55–66. doi: 10.1016/j.molcel.2014.08.019 25242144

[36] Kelly S Greenman C Cook PR Papantonis A. Exon skipping is correlated with exon circularization. J Mol Biol (2015) 427:2414–7. doi: 10.1016/j.jmb.2015.02.018 25728652

[37] Starke S Jost I Rossbach O Schneider T Schreiner S Hung LH. Exon circularization requires canonical splice signals. Cell Rep (2015) 10:103–11. doi: 10.1016/j.celrep.2014.12.002 25543144

[38] Liang D Tatomer DC Luo Z Wu H Yang L Chen LL. The output of protein-coding genes shifts to circular RNAs when the pre-mRNA processing machinery is limiting. Mol Cell (2017) 68:940–54.e3. doi: 10.1016/j.molcel.2017.10.034 29174924 PMC5728686

[39] Noto JJ Schmidt CA Matera AG. Engineering and expressing circular RNAs *via* tRNA splicing. RNA Biol (2017) 14:978–84. doi: 10.1080/15476286.2017.1317911 PMC5680671 28402213

[40] Schmidt CA Giusto JD Bao A Hopper AK Matera AG. Molecular determinants of metazoan tricRNA biogenesis. Nucleic Acids Res (2019) 47:6452–65. doi: 10.1093/nar/gkz311 PMC6614914 31032518

[41] Li Z Huang C Bao C Chen L Lin M Wang X. Exon-intron circular RNAs regulate transcription in the nucleus. Nat Struct Mol Biol (2015) 22:256–64. doi: 10.1038/nsmb.2959 25664725

[42] Zhang Y Zhang XO Chen T Xiang JF Yin QF Xing YH. Circular intronic long noncoding RNAs. Mol Cell (2013) 51:792–806. doi: 10.1016/j.molcel.2013.08.017 24035497

[43] Tan A Li Q Chen L. CircZFR promotes hepatocellular carcinoma progression through regulating miR-3619–5p/CTNNB1 axis and activating wnt/β-catenin pathway. Arch Biochem Biophys (2019) 661:196–202. doi: 10.1016/j.abb.2018.11.020 30468709

[44] Zhu P Liang H Huang X Zeng Q Liu Y Lv J. Circular RNA Hsa_circ_0004018 inhibits wnt/β-catenin signaling pathway by targeting microRNA-626/DKK3 in hepatocellular carcinoma. Onco Targets Ther (2020) 13:9351–64. doi: 10.2147/OTT.S254997 PMC7519839 33061423

[45] Huang XY Huang ZL Zhang PB Huang XY Huang J Wang HC. CircRNA-100338 is associated with mTOR signaling pathway and poor prognosis in hepatocellular carcinoma. Front Oncol (2019) 9:392. doi: 10.3389/fonc.2019.00392 31157168 PMC6528706

[46] Dong W Dai ZH Liu FC Guo XG Ge CM Ding J. The RNA-binding protein RBM3 promotes cell proliferation in hepatocellular carcinoma by regulating circular RNA SCD-circRNA 2 production. EBioMedicine (2019) 45:155–67. doi: 10.1016/j.ebiom.2019.06.030 PMC6642271 31235426

[47] Liu Z Wang Q Wang X Xu Z Wei X Li J. Circular RNA cIARS regulates ferroptosis in HCC cells through interacting with RNA binding protein ALKBH5. Cell Death Discovery (2020) 6:72. doi: 10.1038/s41420-020-00306-x 32802409 PMC7414223

[48] Conn VM Hugouvieux V Nayak A Conos SA Capovilla G Cildir G. A circRNA from SEPALLATA3 regulates splicing of its cognate mRNA through r-loop formation. Nat Plants (2017) 3:17053. doi: 10.1038/nplants.2017.53 28418376

[49] Yang Y Fan X Mao M Song X Wu P Zhang Y. Extensive translation of circular RNAs driven by N6-methyladenosine. Cell Res (2017) 27:626–41. doi: 10.1038/cr.2017.31 PMC5520850 28281539

[50] Yang Y Gao X Zhang M Yan S Sun C Xiao F. Novel role of FBXW7 circular RNA in repressing glioma tumorigenesis. J Natl Cancer Inst (2018) 110:304–15. doi: 10.1093/jnci/djx166 PMC6019044 28903484

[51] Memczak S Jens M Elefsinioti A Torti F Krueger J Rybak A. Circular RNAs are a Large class of animal RNAs with regulatory potency. Nature (2013) 495:333–8. doi: 10.1038/nature11928 23446348

[52] Du WW Fang L Yang W Wu N Awan FM Yang Z. Induction of tumor apoptosis through a circular RNA enhancing Foxo3 activity. Cell Death Differ (2017) 24:357–70. doi: 10.1038/cdd.2016.133 PMC5299715 27886165

[53] Fu X Zhang J He X Yan X Wei J Huang M. Circular RNA MAN2B2 promotes cell proliferation of hepatocellular carcinoma cells *via* the miRNA-217/ MAPK1 axis. J Cancer (2020) 11:3318–26. doi: 10.7150/jca.36500 PMC7097945 32231737

[54] Luo Z Lu L Tang Q Wei W Chen P Chen Y. CircCAMSAP1 promotes hepatocellular carcinoma progression through miR-1294/GRAMD1A pathway. J Cell Mol Med (2021) 25:3793–802. doi: 10.1111/jcmm.16254 PMC8051675 33484498

[55] Cai H Hu B Ji L Ruan X Zheng Z. Hsa_circ_0103809 promotes cell proliferation and inhibits apoptosis in hepatocellular carcinoma by targeting miR-490-5p/SOX2 signaling pathway. Am J Transl Res (2018) 10:1690–702. PMC6038084 30018710

[56] Mittal V. Epithelial mesenchymal transition in tumor metastasis. Annu Rev Pathol (2018) 13:395–412. doi: 10.1146/annurev-pathol-020117-043854 29414248

[57] Pan G Liu Y Shang L Zhou F Yang S. EMT-associated microRNAs and their roles in cancer stemness and drug resistance. Cancer Commun (2021) 41:199–217. doi: 10.1002/cac2.12138 PMC7968884 33506604

[58] Zhang X Luo P Jing W Zhou H Liang C Tu J. circSMAD2 inhibits the epithelial–mesenchymal transition by targeting miR-629 in hepatocellular carcinoma. OncoTargets Ther (2018) 11:2853–63. doi: 10.2147/OTT.S158008 PMC5962255 29844683

[59] Zhu C Su Y Liu L Wang S Liu Y Wu. J. Circular RNA hsa_circ_0004277 stimulates malignant phenotype of hepatocellular carcinoma and epithelial-mesenchymal transition of peripheral cells. Front Cell Dev Biol (2021) 8:585565. doi: 10.3389/fcell.2020.585565 33511111 PMC7835424

[60] Meng J Chen S Han JX Qian B Wang XR Zhong WL. Twist1 regulates vimentin through Cul2 circular RNA to promote EMT in hepatocellular carcinoma. Cancer Res (2018) 78:4150–62. doi: 10.1158/0008-5472.CAN-17-3009 29844124

[61] Xu G Zhang P Liang H Xu Y Shen J Wang W. Circular RNA hsa_circ_0003288 induces EMT and invasion by regulating hsa_circ_0003288/miR-145/PD-L1 axis in hepatocellular carcinoma. Cancer Cell Int (2021) 21:212. doi: 10.1186/s12935-021-01902-2 33858418 PMC8048300

[62] Song LN Qiao GL Yu J Yang CM Chen Y Deng ZF. Hsa_circ_0003998 promotes epithelial to mesenchymal transition of hepatocellular carcinoma by sponging miR-143-3p and PCBP1. J Exp Clin Cancer Res (2020) 39:114. doi: 10.1186/s13046-020-01576-0 32552766 PMC7302140

[63] Jin J Liu H Jin M Li W Xu H Wei F. Silencing of hsa_circ_0101145 reverses the epithelial-mesenchymal transition in hepatocellular carcinoma *via* regulation of the miR-548c-3p/LAMC2 axis. AGING (2020) 12:11623–35. doi: 10.18632/aging.103324 PMC7343517 32554866

[64] Zhang B Zhou J. CircSEC24A (hsa_circ_0003528) interference suppresses epithelial-mesenchymal transition of hepatocellular carcinoma cells *via* miR-421/MMP3 axis. Bioengineered (2022) 13:9050–63. doi: 10.1080/21655979.2022.2057761 PMC9161912 35400271

[65] Feng KL Diao N Zhou ZW Fang CK Wang JN Zhang Y. CircFGGY inhibits cell growth, invasion and epithelial-mesenchymal transition of hepatocellular carcinoma *via* regulating the miR-545-3p/Smad7 axis. Front Cell Dev Biol (2022) 10:850708. doi: 10.3389/fcell.2022.850708 35592246 PMC9110866

[66] Huang G Liang M Liu H Huang J Li P Wang C. CircRNA hsa_circRNA_104348 promotes hepatocellular carcinoma progression through modulating miR-187-3p/RTKN2 axis and activating wnt/β-catenin pathway. Cell Death Dis (2020) 11:1065. doi: 10.1038/s41419-020-03276-1 33311442 PMC7734058

[67] Qiu L Huang Y Li Z Dong X Chen G Xu H. Circular RNA profiling identifies circADAMTS13 as a miR-484 sponge which suppresses cell proliferation in hepatocellular carcinoma. Mol Oncol (2019) 13:441–55. doi: 10.1002/1878-0261.12424 PMC6360375 30537115

[68] Benson AB D'Angelica MI Abbott DE Anaya DA Anders R Are C. Hepatobiliary cancers, version 2.2021, NCCN clinical practice guidelines in oncology. J Natl Compr Canc Netw (2021) 19:541–65. doi: 10.6004/jnccn.2021.0022 34030131

[69] Llovet JM Ricci S Mazzaferro V Hilgard P Gane E Blanc JF. Sorafenib in advanced hepatocellular carcinoma. N Engl J Med (2008) 359:378–90. doi: 10.1056/NEJMoa0708857 18650514

[70] Yang C Dong Z Hong H Dai B Song F Geng L. circFN1 mediates sorafenib resistance of hepatocellular carcinoma cells by sponging miR-1205 and regulating E2F1 expression. Mol Ther Nucleic Acids (2020) 22:421–33. doi: 10.1016/j.omtn.2020.08.039 PMC7533358 33230446

[71] Xu J Wan Z Tang M Lin Z Jiang S Ji L. N6-methyladenosine-modified CircRNA-SORE sustains sorafenib resistance in hepatocellular carcinoma by regulating β-catenin signaling. Mol Cancer (2020) 19:163. doi: 10.1186/s12943-020-01281-8 33222692 PMC7681956

[72] Weng H Zeng L Cao L Chen T Li Y Xu Y. circFOXM1 contributes to sorafenib resistance of hepatocellular carcinoma cells by regulating MECP2 *via* miR-1324. Mol Ther Nucleic Acids (2021) 23:811–20. doi: 10.1016/j.omtn.2020.12.019 PMC7868711 33614231

[73] Wang S Liu D Wei H Hua Y Shi G Qiao J. The hsa_circRNA_102049 mediates the sorafenib sensitivity of hepatocellular carcinoma cells by regulating reelin gene expression. Bioengineered (2022) 13:2272–84. doi: 10.1080/21655979.2021.2024332 PMC8973865 35034536

[74] Huang H Peng J Yi S Ding C Ji W Huang Q. Circular RNA circUBE2D2 functions as an oncogenic factor in hepatocellular carcinoma sorafenib resistance and glycolysis. Am J Transl Res (2021) 13:6076–86. PMC8290719 34306346

[75] Zhang P Sun H Wen P Wang Y Cui Y Wu J. circRNA circMED27 acts as a prognostic factor and mediator to promote lenvatinib resistance of hepatocellular carcinoma. Mol Ther Nucleic Acids (2021) 27:293–303. doi: 10.1016/j.omtn.2021.12.001 35024242 PMC8718824

[76] Huang XY Zhang PF Wei CY Peng R Lu JC Gao C. Circular RNA circMET drives immunosuppression and anti-PD1 therapy resistance in hepatocellular carcinoma *via* the miR-30-5p/snail/DPP4 axis. Mol Cancer (2020) 19:92. doi: 10.1186/s12943-020-01213-6 32430013 PMC7236145

[77] Lu JC Zhang PF Huang XY Guo XJ Gao C Zeng HY. Amplification of spatially isolated adenosine pathway by tumor–macrophage interaction induces anti-PD1 resistance in hepatocellular carcinoma. J Hematol Oncol (2021) 14:1–200. doi: 10.1186/s13045-021-01207-x 34838121 PMC8627086

[78] Li J Qin X Wu R Wan L Zhang L Liu R. Circular RNA circFBXO11 modulates hepatocellular carcinoma progress and oxaliplatin resistance through miR-605/FOXO3/ABCB1 axis. J Cell Mol Med (2020) 24:5152–61. doi: 10.1111/jcmm.15162 PMC7205830 32222024

[79] Fan W Chen L Wu X Zhang T. Circ_0031242 silencing mitigates the progression and drug resistance in DDP-resistant hepatoma cells by the miR-924/POU3F2 axis. Cancer Manage Res (2021) 13:743–55. doi: 10.2147/CMAR.S272851 PMC7847388 33531841

[80] Luo Y Fu Y Huang R Gao M Liu F Gui R. CircRNA_101505 sensitizes hepatocellular carcinoma cells to cisplatin by sponging miR-103 and promotes oxidored-nitro domain-containing protein 1 expression. Cell Death Discovery (2019) 5:121. doi: 10.1038/s41420-019-0202-6 31372241 PMC6662675

[81] Huang W Huang F Feng C. CircFoxo3 promotes adriamycin resistance through regulation of miR-199a-5p/ATP binding cassette subfamily c member 1 axis in hepatocellular carcinoma. Onco Targets Ther (2020) 13:5113–22. doi: 10.2147/OTT.S243571 PMC7292492 32606732

[82] Keir ME Butte MJ Freeman GJ Sharpe AH. PD-1 and its ligands in tolerance and immunity. Annu Rev Immunol (2008) 26:677–704. doi: 10.1146/annurev.immunol.26.021607.090331 18173375 PMC10637733

[83] Zhang XL Xu LL Wang F. Hsa_circ_0020397 regulates colorectal cancer cell viability,apoptosis and invasion by promoting the expression of the miR-138 targets TERT and PD-L1. Cell Biol Int (2017) 41:1056–64. doi: 10.1002/cbin.10826 28707774

[84] DeBerardinis RJ Chandel NS. Fundamentals of cancer metabolism. Sci Adv (2016) 2:e1600200. doi: 10.1126/sciadv.1600200 27386546 PMC4928883

[85] Park JH Pyun WY Park HW. Cancer metabolism: Phenotype, signaling and therapeutic targets. Cells (2020) 9:2308. doi: 10.3390/cells9102308 33081387 PMC7602974

[86] Luengo A Gui DY Vander Heiden MG. Targeting metabolism for cancer therapy. Cell Chem Biol (2017) 24:1161–80. doi: 10.1016/j.chembiol.2017.08.028 PMC5744685 28938091

[87] Li Q Pan X Zhu D Deng Z Jiang R Wang X. Circular RNA MAT2B promotes glycolysis and malignancy of hepatocellular carcinoma through the miR-338-3p/PKM2 axis under hypoxic stress. Hepatology (2019) 70:1298–316. doi: 10.1002/hep.30671 31004447

[88] Chen W Li Y Zhong J Wen G. Circ-PRKCI targets miR-1294 and miR-186-5p by downregulating FOXK1 expression to suppress glycolysis in hepatocellular carcinoma. Mol Med Rep (2021) 23:464. doi: 10.3892/mmr.2021.12103 33880589 PMC8097765

[89] Liu W Yin C Liu Y. Circular RNA circ_0091579 promotes hepatocellular carcinoma proliferation, migration, invasion, and glycolysis through miR-490-5p/CASC3 axis. Cancer Biother Radiopharm (2021) 36:863–78. doi: 10.1089/cbr.2019.3472 32673066

[90] Ding Z Guo L Deng Z Li P. Circ-PRMT5 enhances the proliferation, migration and glycolysis of hepatoma cells by targeting miR-188-5p/HK2 axis. Ann Hepatol (2020) 19:269–79. doi: 10.1016/j.aohep.2020.01.002 32089501

[91] Beyaert R Beaugerie L Van Assche G Brochez L Renauld JC Viguier M. Cancer risk in immune-mediated inflammatory diseases (IMID). Mol Cancer (2013) 12:98. doi: 10.1186/1476-4598-12-98 23987103 PMC3765952

[92] Hauck F Gennery AR Seidel MG. Editorial: The relationship between cancer predisposition and primary immunodeficiency. Front Immunol (2019) 10:1781. doi: 10.3389/fimmu.2019.01781 31417559 PMC6683758

[93] Shi M Li ZY Zhang LM Wu XY Xiang SH Wang YG. Hsa_circ_0007456 regulates the natural killer cell-mediated cytotoxicity toward hepatocellular carcinoma *via* the miR-6852-3p/ICAM-1 axis. Cell Death Dis (2021) 12:94. doi: 10.1038/s41419-020-03334-8 33462208 PMC7814008

[94] Ma Y Zhang C Zhang B Yu H Yu Q. circRNA of AR suppressed PABPC1 91bp enhances the cytotoxicity of natural killer cells against hepatocellular carcinoma *via* upregulating UL16 binding protein 1. Oncol Lett (2019) 17:388–97. doi: 10.3892/ol.2018.9606 PMC6313186 30655779

[95] Zhang PF Gao C Huang XY Lu JC Guo XJ Shi GM. Cancer cell-derived exosomal circUHRF1 induces natural killer cell exhaustion and may cause resistance to anti-PD1 therapy in hepatocellular carcinoma. Mol Cancer (2020) 19:110. doi: 10.1186/s12943-020-01222-5 32593303 PMC7320583

[96] Wang X Sheng W Xu T Xu J Gao R Zhang Z. CircRNA hsa_circ_0110102 inhibited macrophage activation and hepatocellular carcinoma progression *via* miR-580-5p/PPARα/CCL2 pathway. AGING (2021) 13:11969–87. doi: 10.18632/aging.202900 PMC8109088 33891564

[97] Cao P Ma B Sun D Zhang W Qiu J Qin L. hsa_circ_0003410 promotes hepatocellular carcinoma progression by increasing the ratio of M2/M1 macrophages through the miR-139-3p/CCL5 axis. Cancer Sci (2022) 113:634–47. doi: 10.1111/cas.15238 PMC8819332 34890089

[98] Zhang L Zhang J Li P Li T Zhou Z Wu H. Exosomal hsa_circ_0004658 derived from RBPJ overexpressed-macrophages inhibits hepatocellular carcinoma progression *via* miR-499b-5p/JAM3. Cell Death Dis (2022) 13:32. doi: 10.1038/s41419-021-04345-9 35013102 PMC8748962

[99] Tang M Yang M Wu G Mo S Wu X Zhang S. Epigenetic induction of mitochondrial fission is required for maintenance of liver cancer-initiating cells. Cancer Res (2021) 81:3835–48. doi: 10.1158/0008-5472.CAN-21-0436 34049973

[100] Lao VV Grady WM. Epigenetics and colorectal cancer. Nat Rev Gastroenterol Hepatol (2011) 8:686–700. doi: 10.1038/nrgastro.2011.173 22009203 PMC3391545

[101] Sun L Zhang H Gao P. Metabolic reprogramming and epigenetic modifications on the path to cancer. Protein Cell (2022) 13:877–919. doi: 10.1007/s13238-021-00846-7 34050894 PMC9243210

[102] Sun T Wu R Ming L. The role of m6A RNA methylation in cancer. BioMed Pharmacother (2019) 112:108613. doi: 10.1016/j.biopha.2019.108613 30784918

[103] Nombela P Miguel-López B Blanco S. The role of m6A, m5C and Ψ RNA modifications in cancer: Novel therapeutic opportunities. Mol Cancer (2021) 20:18. doi: 10.1186/s12943-020-01263-w 33461542 PMC7812662

[104] Rong D Wu F Lu C Sun G Shi X Chen X. m6A modification of circHPS5 and hepatocellular carcinoma progression through HMGA2 expression. Mol Ther Nucleic Acids (2021) 26:637–48. doi: 10.1016/j.omtn.2021.09.001 PMC8517093 34703649

[105] Wu A Hu Y Xu Y Xu J Wang X Cai A. Methyltransferase-like 3-mediated m6A methylation of Hsa_circ_0058493 accelerates hepatocellular carcinoma progression by binding to YTH domain-containing protein 1. Front Cell Dev Biol (2021) 9:762588. doi: 10.3389/fcell.2021.762588 34888309 PMC8650312

[106] Zhao Z Song J Tang B Fang S Zhang D Zheng L. CircSOD2 induced epigenetic alteration drives hepatocellular carcinoma progression through activating JAK2/STAT3 signaling pathway. J Exp Clin Cancer Res (2020) 39:259. doi: 10.1186/s13046-020-01769-7 33234142 PMC7687771

[107] Du A Li S Zhou Y Disoma C Liao Y Zhang Y. M6A-mediated upregulation of circMDK promotes tumorigenesis and acts as a nanotherapeutic target in hepatocellular carcinoma. Mol Cancer (2022) 21:109. doi: 10.1186/s12943-022-01575-z 35524319 PMC9074191

[108] Wang M Yang Y Yang J Yang J Han S. circ_KIAA1429 accelerates hepatocellular carcinoma advancement through the mechanism of m6A-YTHDF3-Zeb1. Life Sci (2020) 257:118082. doi: 10.1016/j.lfs.2020.118082 32653519

[109] Cai J Chen Z Zhang Y Wang J Zhang Z Wu J. CircRHBDD1 augments metabolic rewiring and restricts immunotherapy efficacy *via* m6A modification in hepatocellular carcinoma. Mol Ther Oncolytics (2022) 24:755–71. doi: 10.1016/j.omto.2022.02.021 PMC8908059 35317519

[110] Chi F Cao Y Chen Y. Analysis and validation of circRNA-miRNA network in regulating m6A RNA methylation modulators reveals CircMAP2K4/ miR-139-5p/YTHDF1Axis involving the proliferation of hepatocellular carcinoma. Front Oncol (2021) 11:560506. doi: 10.3389/fonc.2021.560506 33708621 PMC7940687

[111] Dong ZR Ke AW Li T Cai JB Yang YF Zhou W. CircMEMO1 modulates the promoter methylation and expression of TCF21 to regulate hepatocellular carcinoma progression and sorafenib treatment sensitivity. Mol Cancer (2021) 20:75. doi: 10.1186/s12943-021-01361-3 33985545 PMC8117652

[112] Bahn JH Zhang Q Li F Chan TM Lin X Kim Y. The landscape of microRNA, piwi-interacting RNA, and circular RNA in human saliva. Clin Chem (2015) 61:221–30. doi: 10.1373/clinchem.2014.230433 PMC4332885 25376581

[113] Lei B Zhou J Xuan X Tian Z Zhang M Gao W. Circular RNA expression profiles of peripheral blood mononuclear cells in hepatocellular carcinoma patients by sequence analysis. Cancer Med (2019) 8:1423–33. doi: 10.1002/cam4.2010 PMC6488130 30714679

[114] Liu R Li Y Wu A Kong M Ding W Hu Z. Identification of plasma hsa_circ_0005397 and combined with serum AFP, AFP-L3 as potential biomarkers for hepatocellular carcinoma. Front Pharmacol (2021) 12:639963. doi: 10.3389/fphar.2021.639963 33679420 PMC7933497

[115] Weng Q Chen M Li M Zheng YF Shao G Fan W. Global microarray profiling identified hsa_ circ_0064428 as a potential immune-associated prognosis biomarker for hepatocellular carcinoma. J Med Genet (2019) 56:32–8. doi: 10.1136/jmedgenet-2018-105440 30120213

[116] Li X Yang J Yang X Cao T. Dysregulated circ_0004913, circ_0008160, circ_0000517, and their potential as biomarkers for disease monitoring and prognosis in hepatocellular carcinoma. J Clin Lab Anal (2021) 35:e23785. doi: 10.1002/jcla.23785 34018640 PMC8183933

[117] Sun XH Wang YT Li GF Zhang N Fan L. Serum-derived three-circRNA signature as a diagnostic biomarker for hepatocellular carcinoma. Cancer Cell Int (2020) 20:226. doi: 10.1186/s12935-020-01302-y 32536814 PMC7288432

[118] Yu J Ding WB Wang MC Guo XG Xu J Xu QG. Plasma circular RNA panel to diagnose hepatitis B virus-related hepatocellular carcinoma: A large-scale, multicenter study. Int J Cancer (2020) 146:1754–63. doi: 10.1002/ijc.32647 31456215

[119] Wu C Deng L Zhuo H Chen X Tan Z Han S. Circulating circRNA predicting the occurrence of hepatocellular carcinoma in patients with HBV infection. J Cell Mol Med (2020) 24:10216–22. doi: 10.1111/jcmm.15635 PMC7520265 32692470

[120] Zhang J Chang Y Xu L Qin L. Elevated expression of circular RNA circ_0008450 predicts dismal prognosis in hepatocellular carcinoma and regulates cell proliferation, apoptosis, and invasion *via* sponging miR-548p. J Cell Biochem (2019) 120:9487–94. doi: 10.1002/jcb.28224 30556306

[121] Zhang X Qiu S Luo P Zhou H Jing W Liang C. Down-regulation of hsa_circ_0001649 in hepatocellular carcinoma predicts a poor prognosis. Cancer biomark (2018) 22:135–42. doi: 10.3233/CBM-171109 PMC13098531 29630526

[122] Li W Zhou X Wu X Wei J Huang Z. The role of circular RNA hsa_circ_0085616 in proliferation and migration of hepatocellular carcinoma cells. Cancer Manag Res (2019) 11:7369–76. doi: 10.2147/CMAR.S211020 PMC6689128 31496798

[123] Yu J Xu QG Wang ZG Yang Y Zhang L Ma JZ. Circular RNA cSMARCA5 inhibits growth and metastasis in hepatocellular carcinoma. J Hepatol (2018) 68:1214–27. doi: 10.1016/j.jhep.2018.01.012 29378234

[124] Wang L Long H Zheng Q Bo X Xiao X Li B. Circular RNA circRHOT1 promotes hepatocellular carcinoma progression by initiation of NR2F6 expression. Mol Cancer (2019) 18:119. doi: 10.1186/s12943-019-1046-7 31324186 PMC6639939

[125] Wei Y Chen X Liang C Ling Y Yang X Ye X. A noncoding regulatory RNAs network driven by circ-CDYL acts specifically in the early stages hepatocellular carcinoma. Hepatology (2020) 71:130–47. doi: 10.1002/hep.30795 31148183

[126] Hu ZQ Zhou SL Li J Zhou ZJ Wang PC Xin HY. Circular RNA sequencing identifies CircASAP1 as a key regulator in hepatocellular carcinoma metastasis. Hepatology (2020) 72:906–22. doi: 10.1002/hep.31068 31838741

[127] Zhang X Xu Y Qian Z Zheng W Wu Q Chen Y. circRNA_104075 stimulatesYAP-dependent tumorigenesis through the regulation of HNF4a and may serve as a diagnostic marker in hepatocellular carcinoma. Cell Death Dis (2018) 9:1901. doi: 10.1038/s41419-018-1132-6 PMC6202383 30361504

[128] Sun S Wang W Luo X Li Y Liu B Li X. Circular RNA circ-ADD3 inhibits hepatocellular carcinoma metastasis through facilitating EZH2 degradation *via* CDK1-mediated ubiquitination. Am J Cancer Res (2019) 9:1695–707. PMC6726993 31497351

[129] Matboli M Shafei AE Ali MA Ashry AM Kamal KM Agag MA. circRNAs (hsa_circ_00156, hsa_circ _000224, and hsa_circ _000520) are novel potential biomarkers in hepatocellular carcinoma. J Cell Biochem (2018) 120:7711–24. doi: 10.1002/jcb.28045 30426540

[130] Wang X Wang X Li W Zhang Q Chen J Chen T. Up-regulation of hsa_circ_0000517 predicts adverse prognosis of hepatocellular carcinoma. Front Oncol (2019) 9:1105. doi: 10.3389/fonc.2019.01105 31750237 PMC6842961

[131] Jiang Z Shen L Wang S Wu S Hu Y Guo J. Hsa_circ_0028502 and hsa_circ_0076251 are potential novel biomarkers for hepatocellular carcinoma. Cancer Med (2019) 8:7278–87. doi: 10.1002/cam4.2584 PMC6885881 31595711

[132] Qiao GL Chen L Jiang WH Yang C Yang CM Song LN. Hsa_circ_0003998 may be used as a new biomarker for the diagnosis and prognosis of hepatocellular carcinoma. Onco Targets Ther (2019) 12:5849–60. doi: 10.2147/OTT.S210363 PMC6650091 31410028

[133] Chen D Zhang C Lin J Song X Wang H. Screening differential circular RNA expression profiles reveal that hsa_circ_0128298 is a biomarker in the diagnosis and prognosis of hepatocellular carcinoma. Cancer Manag Res (2018) 10:1275–83. doi: 10.2147/CMAR.S166740 PMC5965387 29849467

[134] Li Z Zhou Y Yang G He S Qiu X Zhang L. Using circular RNA SMARCA5 as a potential novel biomarker for hepatocellular carcinoma. Clin Chim Acta (2019) 492:37–44. doi: 10.1016/j.cca.2019.02.001 30716279

[135] Zhang X Zhou H Jing W Luo P Qiu S Liu X. The circular RNA hsa_circ_0001445 regulates the proliferation and migration of hepatocellular carcinoma and may serve as a diagnostic biomarker. Dis Markers (2018) 2018:3073467. doi: 10.1155/2018/3073467 29785229 PMC5896272

[136] Kou P Zhang C Lin J Wang H. Circular RNA hsa_circ_0078602 may have potential as a prognostic biomarker for patients with hepatocellular carcinoma. Oncol Lett (2019) 17:2091–8. doi: 10.3892/ol.2018.9863 PMC6341904 30675276

[137] Zhou S Wei J Wang Y Liu X. Cisplatin resistance associated circRNA_101237 serves as a prognostic biomarker in hepatocellular carcinoma. Exp Ther Med (2020) 19:2733–40. doi: 10.3892/etm.2020.8526 PMC7092923 32226487

[138] Duan JL Chen W Xie JJ Zhang ML Nie RC Liang H. A novel peptide encoded by N6-methyladenosine modified circMAP3K4 prevents apoptosis in hepatocellular carcinoma. Mol Cancer (2022) 21:93. doi: 10.1186/s12943-022-01537-5 35366894 PMC8976336

[139] Li D Zhang J Yang J Wang J Zhang R Li J. CircMTO1 suppresses hepatocellular carcinoma progression *via* the miR-541-5p/ZIC1 axis by regulating wnt/β-catenin signaling pathway and epithelial-to-mesenchymal transition. Cell Death Dis (2021) 13:12. doi: 10.1038/s41419-021-04464-3 34930906 PMC8688446

[140] Pu J Wang J Li W Lu Y Wu X Long X. hsa_circ_0000092 promotes hepatocellular carcinoma progression through up-regulating HN1 expression by binding to microRNA-338-3p. J Cell Mol Med (2020). doi: 10.1111/jcmm.15010 PMC10941524 32077624

[141] Liu W Pan Y Zhu H Zhou Y Zhang H Liu L. CircRNA_0008194 functions as a ceRNA to promote invasion of hepatocellular carcinoma *via* inhibiting miR-190a/AHNAK signaling pathway. J Clin Lab Anal (2022) 36:e24286. doi: 10.1002/jcla.24286 35199873 PMC8993631

[142] Liu Z Yu Y Huang Z Kong Y Hu X Xiao W. CircRNA-5692 inhibits the progression of hepatocellular carcinoma by sponging miR-328-5p to enhance DAB2IP expression. Cell Death Dis (2019) 10:.900. doi: 10.1038/s41419-019-2089-9 31776329 PMC6881381

[143] Gu Y Wang Y He L Zhang J Zhu X Liu N. Circular RNA circIPO11 drives self-renewal of liver cancer initiating cells *via* hedgehog signaling. Mol Cancer (2021) 20:132. doi: 10.1186/s12943-021-01435-2 34649567 PMC8515748

[144] Huang XY Huang ZL Huang J Xu B Huang XY Xu YH. Exosomal circRNA-100338 promotes hepatocellular carcinoma metastasis *via* enhancing invasiveness and angiogenesis. J Exp Clinl Cancer Res (2020) 39:20. doi: 10.1186/s13046-020-1529-9 PMC6979009 31973767

[145] Li S Li X Xue W Zhang L Yang LZ Cao SM. Screening for functional circular RNAs using the CRISPR-Cas13 system. Nat Methods (2021) 18:51–9. doi: 10.1038/s41592-020-01011-4 33288960

[146] Schultz N Marenstein DR De Angelis DA Wang WQ Nelander S Jacobsen A. Off-target effects dominate a large-scale RNAi screen for modulators of the TGF-β pathway and reveal microRNA regulation of TGFBR2. Silence (2011) 2:3. doi: 10.1186/1758-907X-2-3 21401928 PMC3068080

[147] He AT Liu J Li F Yang BB. Targeting circular RNAs as a therapeutic approach: current strategies and challenges. Signal Transduct Target Ther (2021) 6:185. doi: 10.1038/s41392-021-00569-5 34016945 PMC8137869

[148] Chen YG Kim MV Chen X Batista PJ Aoyama S Wilusz JE. Sensing self and foreign circular RNAs by intron identity. Mol Cell (2017) 67:228–38.e5. doi: 10.1016/j.molcel.2017.05.022 28625551 PMC5610545

[149] Cheng X Tian P Zheng W Yan X. Piplartine attenuates the proliferation of hepatocellular carcinoma cells *via* regulating hsa_circ_100338 expression. Cancer Med (2020) 9:4265–73. doi: 10.1002/cam4.3043 PMC7300402 32281302

[150] Si H Wang H Xiao H Fang Y Wu Z. Anti-tumor effect of celastrol on hepatocellular carcinoma by the circ_SLIT3/miR-223-3p/CXCR4 axis. Cancer Manag Res (2021) 13:1099–111. doi: 10.2147/CMAR.S278023 PMC7872924 33574707

[151] Lin S Zhuang J Zhu L Jiang Z. Matrine inhibits cell growth, migration, invasion and promotes autophagy in hepatocellular carcinoma by regulation of circ_0027345/miR-345-5p/HOXD3 axis. Cancer Cell Int (2020) 20:246. doi: 10.1186/s12935-020-01293-w 32549793 PMC7296946

[152] Bao S Jin S Wang C Tu P Hu K Lu J. Androgen receptor suppresses vasculogenic mimicry in hepatocellular carcinoma *via* circRNA7/miRNA7-5p/VE-cadherin/Notch4 signalling. J Cell Mol Med (2020) 24:14110–20. doi: 10.1111/jcmm.16022 PMC7754040 33118329

[153] Di Timoteo G Dattilo D Centrón-Broco A Colantoni A Guarnacci M Rossi F. Modulation of circRNA metabolism by m6A modification. Cell Rep (2020) 31:107641. doi: 10.1016/j.celrep.2020.107641 32402287

[154] Glažar P Papavasileiou P Rajewsky N. circBase: a database for circular RNAs. RNA (2014) 20:1666–70. doi: 10.1261/rna.043687.113 PMC4201819 25234927

[155] Du Q Han J Gao S Zhang S Pan Y. Hypoxia-induced circular RNA hsa_circ_0008450 accelerates hepatocellular cancer progression *via* the miR-431/AKAP1 axis. Oncol Lett (2020) 20:388. doi: 10.3892/ol.2020.12251 33193848 PMC7656113

[156] Lin T Dai Y Guo X Chen W Zhao J Cao L. Silencing of hsa_circ_0008450 represses hepatocellular carcinoma progression through regulation of microRNA-214-3p/EZH2 axis. Cancer Manag Res (2019) 11:9133–43. doi: 10.2147/CMAR.S222716 PMC6817349 31695501

[157] Tang X Ren H Guo M Qian J Yang Y Gu C. Review on circular RNAs and new insights into their roles in cancer. Comput Struct Biotechnol J (2021) 19:910–28. doi: 10.1016/j.csbj.2021.01.018 PMC7851342 33598105

[158] Li R Jiang J Shi H Qian H Zhang X Xu W. CircRNA: a rising star in gastric cancer. Cell Mol Life Sci (2020) 77:1661–80. doi: 10.1007/s00018-019-03345-5 PMC11104848 31659415

