# Corrigendum: Review of novel functions and implications of circular RNAs in hepatocellular carcinoma

**DOI:** 10.3389/fonc.2024.1380699

**Published:** 2024-02-26

**Authors:** Zheng Liu, Fangming Yang, Zhun Xiao, Yuexuan Liu

**Affiliations:** ^1^ Department of Combination of Traditional Chinese Medicine and Western Medicine, School of Medicine, Henan University of Chinese Medicine, Zhengzhou, China; ^2^ Department of Digestive Diseases, The First Affiliated Hospital of Henan University of Chinese Medicine, Zhengzhou, China

**Keywords:** circular RNAs, hepatocellular carcinoma, functions, implications, drug resistance, epigenetic modifications

Incorrect Funding

In the published article, there was an error in the Funding statement. The funding statement for the National Natural Science Foundation of China was incorrectly displayed as “82104772 and 82205021”. The correct Funding statement appears below.

